# Cooperative short- and long-range interactions enable robust symmetry breaking and axis formation

**DOI:** 10.1101/2025.09.27.678924

**Published:** 2025-09-29

**Authors:** Guoye Guan, Suxuan Wang, T. Glenn Shields, Seong Ho Pahng, Claire Xinyu Shao, Juns Ye, Christoph Budjan, Sahand Hormoz

**Affiliations:** 1 Department of Systems Biology, Harvard Medical School, Boston, MA 02115, USA; 2 Department of Data Science, Dana-Farber Cancer Institute, Boston, MA 02215, USA; 3 Harvard College, Faculty of Arts and Sciences, Harvard University, Boston, MA 02138, USA; 4 Department of Molecular and Cellular Biology, Harvard University, Boston, MA 02138, USA; 5 Department of Chemistry and Chemical Biology, Harvard University, Boston, MA 02138, USA; 6 Boston Latin School, Boston, MA 02115, USA; 7 Broad Institute of MIT and Harvard, Boston, MA 02142, USA; 8 Lead Contact

## Abstract

The establishment of the anterior–posterior (A–P) axis is the first symmetry-breaking event in mammalian development, transforming initially uniform cell populations into a polarized body plan. Gastruloids, aggregates of embryonic stem cells, recapitulate this transition by reproducibly forming a posterior primitive-streak–like pole. To investigate the underlying physical principles, we constructed a coarse-grained agent-based model representing two radially differentiated cell populations — outer/peripheral and inner/core — interacting via short-range adhesion/surface tension and optional long-range, chemotaxis-like forces. Systematic exploration of this morphogenetic landscape revealed that adhesion alone cannot robustly generate a single axis, often leading to weak or unstable asymmetries. By contrast, introducing long-range attraction among peripheral cells markedly broadened the parameter space for robust symmetry breaking, yielding high morphological asymmetry with minimal cell loss. We further implement a minimal, modular gene regulatory network that partitions cells into outer vs. inner states and gates adhesion and peripheral long-range attraction, converting an inside–outside bias into a stable axis. To facilitate further exploration, we developed *DevSim*, a user-friendly platform for simulating coupled genetic–mechanical rules in multicellular systems. Our results suggest that cooperative short- and long-range interactions are necessary design principles for reliable A–P axis formation in gastruloids and provide a framework for dissecting and engineering self-organizing developmental systems.

## INTRODUCTION

Breaking symmetry to establish the anterior–posterior (A–P) axis is a defining event in mammalian development. During gastrulation, a near-uniform epiblast becomes patterned into germ layers and a polarized body axis, with the posterior pole corresponding to the primitive streak and tailbud. Stem cell–derived embryo models (“gastruloids”) provide accessible systems to study this transition: starting from spherical aggregates, both mouse and human gastruloids reproducibly elongate and polarize, with a posterior domain marked by primitive-streak reporters [Beccari et al. Nature 2018; Moris et al. Nature 2020a]. Thus, in vitro models capture core features of A–P patterning in the absence of extraembryonic tissues.

Recent work indicates that A–P symmetry breaking in gastruloids emerges from an inside–out radial asymmetry that is subsequently converted into axial polarity. Imaging and single-cell transcriptomics show that peripheral cells preferentially acquire primitive-streak-like fates and high Wnt activity, whereas core cells remain pluripotent with elevated Nodal signaling [Suppinger et al. Cell Stem Cell 2023; McNamara et al. Nat. Cell Biol. 2024; Dias et al. bioRxiv 2025]. This mutually antagonistic Wnt/Nodal landscape seeds the divergence of outer and inner populations, which later segregate toward opposite poles. Adhesion differences contribute—cadherin switching and differential adhesion gene expression correlate with polarization [Mayran et al. bioRxiv 2023; McNamara et al. Nat. Cell Biol. 2024] — yet adhesion alone often fails: synthetic adhesion-based assemblies tend to multi-cluster or form unstable configurations rather than a single global axis [Toda et al. Science 2018; Stevens et al. Nature 2023; Yamada et al. Cell 2025]. This poses a mechanistic question: what interaction architecture makes axis selection robust in gastruloids?

To answer this, we construct a scalable coarse-grained agent model with two pre-differentiated radial populations (outer/peripheral and inner/core) interacting via (i) short-range adhesion/surface-tension forces and (ii) long-range chemotaxis-like interactions, and we systematically map the design space. A quantitative morphological asymmetry statistic (center-of-mass separation normalized by size) defines the morphogenetic landscape across interaction architectures and strengths. We find that differential adhesion alone does not robustly produce symmetry breaking, whereas adding long-range attraction among peripheral cells dramatically enlarges the parameter region that yields a single, stable axis. Finally, we implement a minimal, modular gene regulatory network—comprising a timer, a transient mutual-inhibition bifurcation, and a maintenance “lock-in”—that maps gene states to mechanical couplings (adhesion; chemotaxis-like attraction). This GRN reproduces inside–outside fate partitioning and drives axis formation by activating peripheral long-range attraction. These results suggest that coupled short- and long-range interactions help explain the reproducible A–P symmetry breaking of mammalian gastruloids, and we provide a flexible simulation platform (*DevSim*) for exploring the underlying genetic–mechanical design principles.

## RESULTS

### Self-organized symmetry breaking robustly emerges in 3D human gastruloids cultured in suspension.

**Figure 1.**
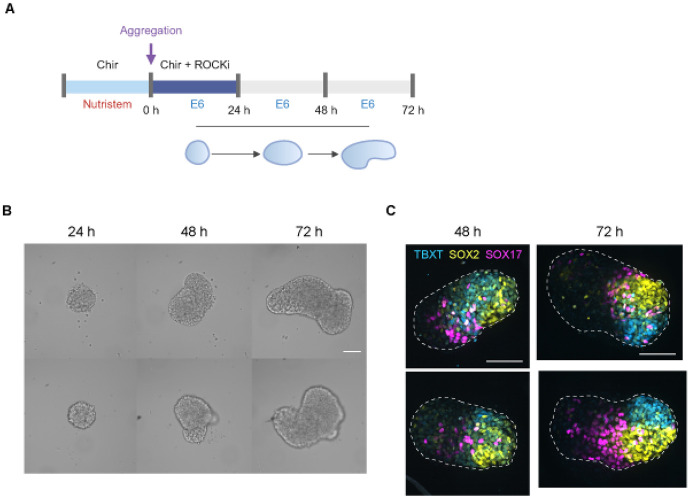
Human stem-cell derived 3D gastruloids exhibit robust symmetry breaking. (A) Experimental schema of the human gastruloid protocol. Human ES cells were pre-treated with the WNT agonist Chir 24h prior to aggregation. To generate gastruloids from pre-treated hES cells, cells were dissociated into single cells and 400 cells were deposited into each 96-well U-bottom multiwell plate in the presence of Chir and ROCKi. On subsequent days until 72h, fresh E6 basal media was added to each well without the addition of factors. Chir, Chiron (CHIR99021); E6, Essential 6 medium; ROCKi, ROCK inhibitor. (B) Representative brightfield images of 2 gastruloid replicates taken every 24h, demonstrating symmetry breaking. (C) Fluorescent images of gastruloids derived from a germ layer reporter hES cell line (RUES2-GLR) reveals organized asymmetric establishment of the primary germ layers in gastruloids between 48-72h. TBXT/BRA, mesoderm, SOX2, neuro-ectoderm, SOX17, endoderm. Scale bar: 100 μm.

To first establish the experimental basis for our theoretical work, we asked whether human gastruloids reproducibly undergo symmetry breaking under defined culture conditions. To this end, we applied a recently established protocol for generating gastruloids from human embryonic stem cells (hESCs) [Moris et al. Nature 2020a; Moris et al. Res. Sq. 2020b]. Approximately 400 cells were aggregated in round-bottom wells and stimulated with the Wnt agonist CHIR99021 (Chir) to induce gastruloid formation (Figure 1A) . While control aggregates without Wnt activation remained spherical, Chir stimulated aggregates reproducibly elongated across and within independent experimental batches (Figure 1B, Figure S1, Movie S1).

At the molecular level, symmetry breaking was also evident. Markers of posterior identity localized to the extending distal pole of the gastruloid, including TBXT/BRA (primitive streak/mesoderm), and SOX2 (neuroectoderm) (Figure 1C). SOX17^+^ (endoderm) cells were initially more dispersed. By 72 h, these progenitor populations had segregated into distinct and adjacent TBXT^+^, SOX2^+^, and SOX17^+^ domains at the distal pole. These findings highlight the reproducibility and robustness of symmetry breaking in human gastruloids, in sharp contrast to the uncontrolled variability in node number recently reported for synthetic organoids that rely solely on differential adhesion [Toda et al. Science 2018; Yamada et al. Cell 2025].

### Searching mechanical architectures and force strengths capable of two-cell-type symmetry breaking.

Both Chir-treated human and mouse 3D gastruloids undergo symmetry breaking from an initially spherical aggregate, where certain gene expression (e.g., ectoderm marker SOX2 [Suppinger et al. Cell Stem Cell 2023; Mayran et al. bioRxiv 2023] and bifurcated Wnt and Nodal signaling activities [McNamara et al. Nat. Cell Biol. 2024; Dias et al. bioRxiv 2025]) change levels along the radial direction from the inside of the sphere to the outside . These observations reveal that inner cells remain more pluripotent, whereas outer cells acquire primitive-streak-like features. Importantly, high Wnt and Nodal signaling activities occupy the outer and inner regions of the aggregate respectively, and are mutually antagonistic, as shown by signaling perturbation experiments [McNamara et al. Nat. Cell Biol. 2024; Dias et al. bioRxiv 2025]. Once symmetry breaking occurs, the two bifurcated cell populations retain their identities and segregate toward the posterior and anterior poles [McNamara et al. Nat. Cell Biol. 2024].

**Figure 2.**
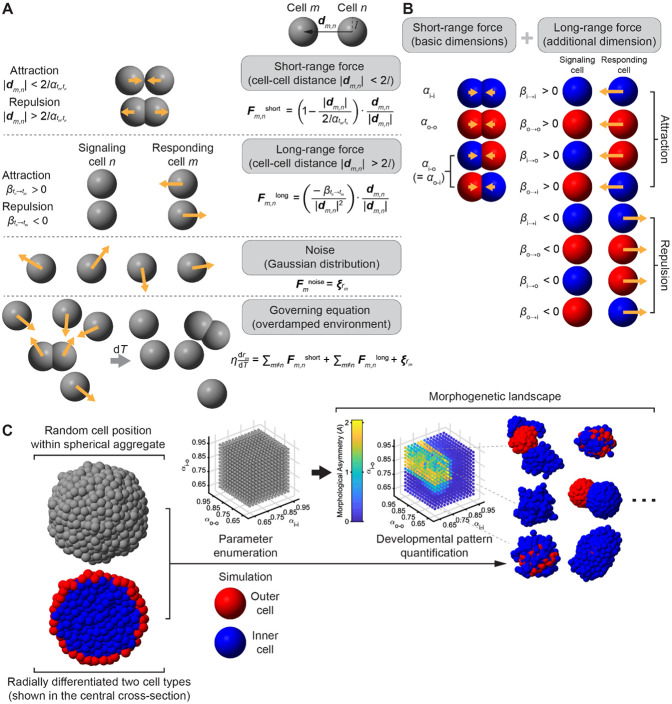
Two-population agent model, interaction channels, and design-space workflow. (A) Model mechanics. Cells are point agents in an overdamped medium. Pairwise short-range contact forces (effective adhesion/surface tension) are indexed by the types of the two cells — outer (o) or inner (i) — not by cell identity: $\alpha_{t_{m},t_{n}}$ with t∈{o,i}. Thus the short-range parameter table is $\left\{\alpha_{i-i},\alpha_{o-o},\alpha_{i-o}=\alpha_{o-i}\right\}$. Beyond the contact range, an optional long-range, chemotaxis-like force acts with strength $\beta_{t_{n}\to t_{m}}$, which depends on the signaling cell’s type ($t_{n}$) and the responding cell’s type ($t_{m}$); β>0 denotes attraction and β<0 repulsion. Stochastic perturbations are Gaussian. (B) Interaction channels. Short-range interactions provide the baseline (“basic dimensions”) via $\alpha_{i-i}$, $\alpha_{o-o}$, and the cross-type term $\alpha_{i-o}=\alpha_{o-i}$. Long-range interactions add four directed channels (“additional dimension”): inner→inner ($\beta_{i\to i}$), outer→outer ($\beta_{o\to o}$), inner→outer ($\beta_{i\to o}$), and outer→inner ($\beta_{o\to i}$). Rows illustrate attraction vs. repulsion for each channel (signaling cell at left, responding cell at right; red = outer, blue = inner). (C) Simulation pipeline and readouts. Aggregates are initialized as near-spherical with a radial prepattern (outer/peripheral shell in red; inner/core in blue) (Figure S2). We enumerate a grid of $\alpha_{t_{m},t_{n}}$ values and, in separate scans, enable individual $\beta_{t_{n}\to t_{m}}$ channels; each setting is simulated over a long duration enough for clear pattern formation (time step length ΔT=0.2; total time duration $T_{total}=150$; for each time step, Gaussian noise with a mean and standard deviation of $\sqrt{\Delta t}\kappa_{M}=\sqrt{0.2}\cdot 0.1$, unless otherwise specified, is added to the cell’s position in all three orthogonal directions). The resulting morphogenetic landscape is summarized by a morphological asymmetry score A (center-of-mass separation of outer vs. inner populations, normalized by size) and illustrated with representative 3D final configurations. Parameter ranges, grid resolution, and noise levels are indicated in the panels.

Provided these experimental insights, we devised a coarse-grained, agent-based mechanical model to capture multicellular interactions (Figure 2). Considering that a 3D human gastruloid contains on the order of a thousand cells during symmetry breaking, we represent each cell as a single point. This provides the simplest and most computationally efficient framework for simulating large-scale cell populations while preserving scalability for additional regulatory layers. This type of minimal multicellular modeling has previously revealed fundamental principles of patterning in embryogenesis and organogenesis [Fickentscher et al. Phys. Rev. Lett. 2016; Nissen et al. PLoS Biol. 2017].

Here, we initiate an *in silico* cell population randomly positioned and contacting each other with homogenous adhesion. The *in silico* cell number is set as 1500 to match the experimentally-measured cell number at 48-h human gastruloid [Moris et al. Nature 2020a; Moris et al. Res. Sq. 2020b], with 25% of them assigned as outer cell type (marked as “o”) and the remaining ones as inner cell type (marked as “i”). The interactive force between any two cell types could be repulsive (volume effect) and attractive (mediated by adhesion) in short range or chemotaxis-like in long range (Figure 2A) [Petridou et al. Cell 2021; Pani et al. *eLife* 2018]. For short-range force, given that E-cadherin has been shown to play a role in symmetry breaking in mammalian gastruloids [Mayran et al. bioRxiv 2023; McNamara et al. Nat. Cell Biol. 2024], we employed an empirically fitted linear force law derived from E-cadherin-mediated adhesion in worm development, which successfully describes differential-adhesion-driven pattern formation [Yamamoto et al. *Development* 2017; Guan et al. *Commun. Nonlinear Sci. Numer. Simul.* 2022]; in this formulation, a parameter α denotes the balance distance (zero net force) between two cells — smaller values denote stronger adhesion, and larger values denote weaker adhesion. For long-range forces, we implemented a phenomenological gravitation-like interaction between cells, parameterized by β — negative values denote repulsion, positive values denote attraction, and zero denotes absence of long-range force. The resulting morphogenetic landscape can thus be described by basic ternary adhesion combinations ($\alpha_{i-i}$, $\alpha_{o-o}$, $\alpha_{i-o}$) and optionally extended with long-range forces $\beta_{i-i}$, $\beta_{o-o}$, $\beta_{i-o}$, $\beta_{o-i}$ or their combinations (Figure 2B).

To systematically search for design principles, we adopted a minimalist strategy commonly used in systems biology [Ma et al. Cell 2009; Chau et al. Cell 2012]: parameters are added only when simpler configurations fail to reproduce the desired outcome (Figure 2C). Accordingly, we first explored the adhesion-only system before introducing additional long-range interactions.

### Differential adhesion is insufficient for robust symmetry breaking.

**Figure 3.**
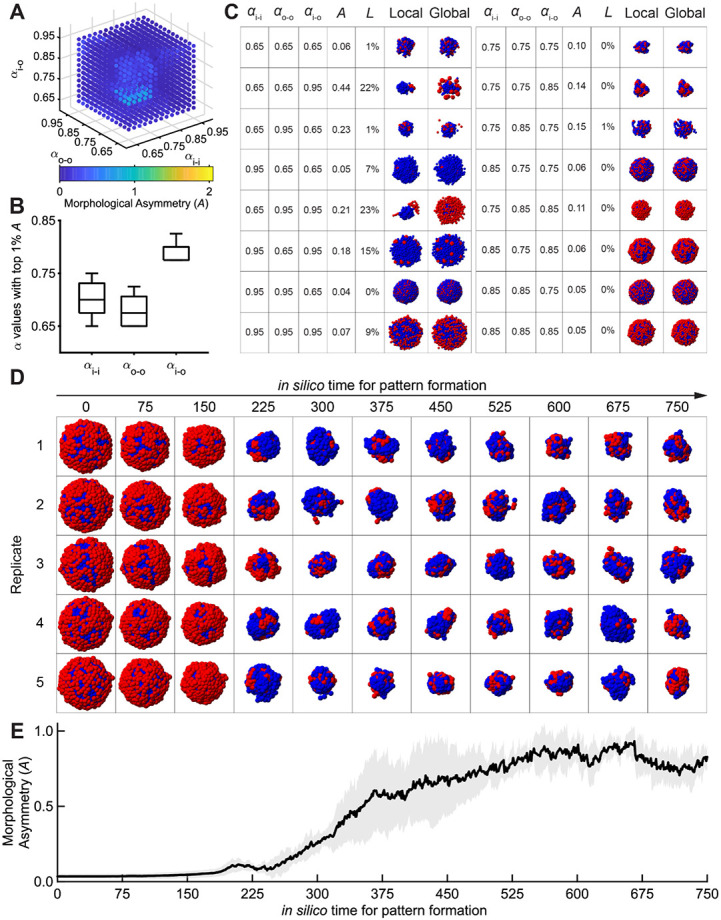
Morphogenetic landscape consisting of short-range force parameters ($\alpha_{i-i}$, $\alpha_{o-o}$, $\alpha_{i-o}$) reveals low morphological asymmetry (A). (A) Heatmap showing morphological asymmetry (A) across the three short-range force parameters ($\alpha_{i-i}$, $\alpha_{o-o}$, $\alpha_{i-o}$) (blue: low A; yellow: high A), revealing maximum A=0.828. (B) Boxplot showing the α value distribution for parameter combinations within the top 1% of A. (C) Representative final morphologies evolved from a spherical aggregate, when the three short-range force parameters ($\alpha_{i-i}$, $\alpha_{o-o}$, $\alpha_{i-o}$) are set as regular values. Results with extreme α values (0.65, mimicking strong adhesion; 0.95, mimicking weak adhesion) are shown on the left; results with moderate α values (0.75 and 0.85, mimicking moderate adhesion) are shown on the right. Here, the local pattern shows the largest aggregate with the most contacting cells; the global pattern shows the entirety of cells within the simulated system. (D) Morphological evolution from a spherical aggregate to weak symmetry breaking. Shown are five independent replicates with the highest morphological asymmetry observed under adhesion parameters $\left(\alpha_{i-i},\alpha_{o-o},\alpha_{i-o}\right)=(0.750,0.725,0.800)$. (E) Morphological asymmetry as a function of *in silico* time, plotted from the five independent replicates with the highest morphological asymmetry observed under short-range force parameters $\left(\alpha_{i-i},\alpha_{o-o},\alpha_{i-o}\right)=(0.750,0.725,0.800)$. Black solid line: mean; gray shade: standard deviation.

For each parameter setting, we ran and repeated the simulation five times with independent noise seeds. We first identified the largest aggregate of contacting cells, and defined cell loss score L as the ratio of the remaining cells to the total population — at the final time point and averaged across runs; we then computed the morphological asymmetry score A averaged over those aggregates — defined as the separation between the centers of mass of the outer-cell and inner-cell populations, normalized by aggregate size [QUANTITATIVE AND STATISTICAL ANALYSIS — Developmental Pattern Description]. Systematic enumeration of the three intercellular adhesion parameters ($\alpha_{i-i}$, $\alpha_{o-o}$, $\alpha_{i-o}$) defines a morphogenetic landscape (Figure 3AC), which exhibits a low morphological asymmetry A (always below 1) even when the inner and outer cell types each maintain stronger adhesion within their own type than between them (Figure 3BD). This observation is consistent with recent synthetic organoid studies [Toda et al. Science 2018; Yamada et al. Cell 2025], where similar adhesion programming for two cell types failed to produce robust symmetry breaking. To further demonstrate that this conclusion is independent of the arbitrarily chosen noise level, we varied both the magnitude and interval of noise. When noise is too low, cells become trapped in local energy minima, preventing cluster formation; conversely, when noise is too high, clusters disintegrate because adhesion forces are insufficient to retain the cells (Figure S3). These simulations suggest that while differential adhesion can initiate symmetry breaking, it alone does not guarantee considerable robustness or performance.

### Peripheral long-range attraction produces robust symmetry breaking.

**Figure 4.**
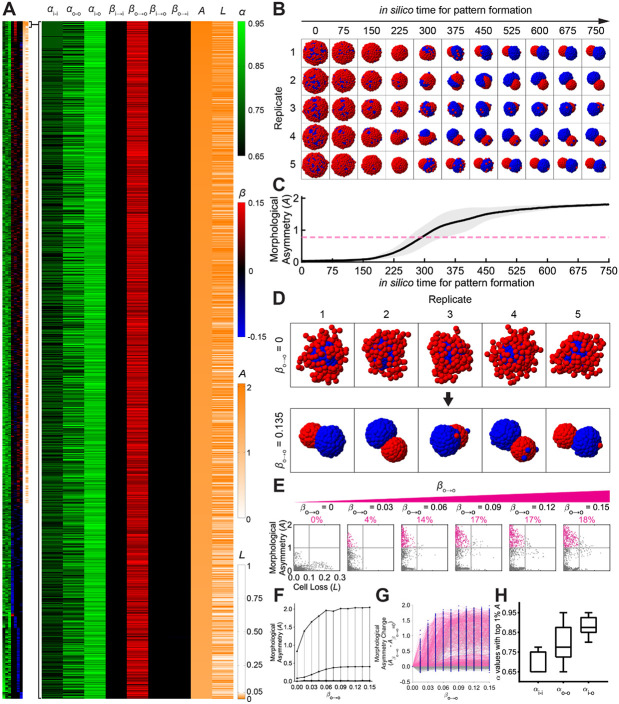
Long-range attraction ($\beta_{o\to o}>0$) among peripheral cell type effectively increases morphological asymmetry (A) and decreases cell loss (L), leading to robust symmetry breaking. (A) Heatmap showing short-range force parameters (Columns 1-3: $\alpha_{i-i}$, $\alpha_{o-o}$, $\alpha_{i-o}$; black: low α representing strong adhesion; green: high , representing weak adhesion), long-range force parameters (Columns 4-7: $\beta_{i\to i}$, $\beta_{o\to o}$, $\beta_{i\to o}$, $\beta_{o\to i}$; black: no interaction; red: high β representing attraction; blue: high β representing repulsion), morphological asymmetry (Column 8: A; white: low A; orange: high A), and cell loss (Column 9: L; white: low L, orange: high L). The complete parameter landscape with 177,957 combinations is displayed on the left, ranked by morphological asymmetry level; the top 1% of conditions with the highest morphological asymmetry are enlarged on the right, highlighting the dominant role of long-range attraction (Column 5: red) in driving symmetry breaking. (B) Morphological evolution from a spherical aggregate to robust symmetry breaking. Shown are five independent replicates with the highest 1% morphological asymmetry. (C) Morphological asymmetry curve over *in silico* time, plotted from the five independent replicates with the highest 1% morphological asymmetry. Black solid line: mean; gray shade: standard deviation; pink dashed line: the maximum final A value when β=0, corresponding to Figure 3. (D) Final morphologies identical to those in (B) and (C), shown across five independent replicates without (top) and with (bottom) long-range attraction. (E) Distribution of short-range force parameter combinations that produce considerable morphological asymmetry (A>1) and cell loss (L<0.1). As long-range attraction $\beta_{o\to o}$ increases, the feasible short-range force parameter combination ($\alpha_{i-i}$, $\alpha_{o-o}$, $\alpha_{i-o}$) region continuously expands from null. (F) Absolute increase in average and maximum morphological asymmetry ($A_{ave}$ and $A_{max}$) over long-range attraction $\beta_{o\to o}$, regarding all the short-range force parameter combinations ($\alpha_{i-i}$, $\alpha_{o-o}$, $\alpha_{i-o}$). (G) Relative increase in morphological asymmetry (A) over long-range attraction $\beta_{o\to o}$, for specific short-range force parameter combination ($\alpha_{i-i}$, $\alpha_{o-o}$, $\alpha_{i-o}$) (connected by gray lines), showing 58.6% of the parameter combinations with consistently higher asymmetry under long-range attraction. (H) Boxplot showing the α value distribution for parameter combinations within the top 1% of A.

We next probed long-range interactions one at a time: for each of the eight possibilities ((i→i, o→o, i→o, o→i) × {attractive, repulsive}), we set only that term to a nonzero value (all other β=0) and exhaustively swept the adhesion parameters ($\alpha_{i-i}$, $\alpha_{o-o}$, $\alpha_{i-o}$) across their full grid. The complete morphogenetic landscape (Figures S4-S11), encompassing 177,957 simulation points, revealed morphological asymmetry values, A, as high as 2.047. Ranking the parameter values by A reveals a striking pattern: long-range attraction among outer cells ($\beta_{o\to o}>0$) consistently appears in the top-scoring solutions and yields two well-separated inner/outer clusters with a single global axis (Figure 4A-D). Within the top 1% of simulations, 99.94% of parameter sets included long-range attraction within outer cells ($\beta_{o\to o}>0$). As $\beta_{o\to o}$ increases from 0, the feasible ($\alpha_{i-i}$, $\alpha_{o-o}$, $\alpha_{i-o}$) region for robust symmetry breaking (defined as A>1 and L<0.1) expands monotonically from empty to nearly 18% of the whole parameter space (Figure 4E); meanwhile, both mean and maximum A rise and then saturate (Figure 4F). For most adhesion settings, turning on $\beta_{o\to o}$ strictly increases A (Figure 4G). In effect, $\beta_{o\to o}$ acts as a global ordering knob that converts a narrow, adhesion-fine-tuned regime into a broad basin of attraction for robust single-axis symmetry breaking.

### Cooperative roles of long- and short-range interactions

Top-performing designs (the top 1% of designs with the highest morphological asymmetry) share a consistent pattern of adhesion (Figure 4H): strong inner–inner adhesion (low $\alpha_{i-i}=0.7154\pm 0.0502$) to prevent fragmentation of the core; weak inner–outer adhesion (high $\alpha_{i-o}=0.8782\pm 0.0422$) to prevent merging of clusters; and moderate outer–outer adhesion (moderate $\alpha_{o-o}=0.7898\pm 0.0912$) to permit reorganization of the periphery during elongation. Long-range outer–outer attraction then coherently pulls the periphery into a polarized architecture while the core remains cohesive, jointly producing robust symmetry breaking. Critically, turning on $\beta_{o\to o}$ also expands the admissible adhesion region for symmetry breaking: the set of ($\alpha_{i-i}$, $\alpha_{o-o}$, $\alpha_{i-o}$) values that yield high A widens markedly, and o-o no longer needs to be “strong” — moderate outer–outer adhesion becomes both permitted and often optimal. In effect, long-range peripheral attraction cooperates with adhesion by relaxing fine-tuning requirements and converting a narrow adhesion-only window into a broad basin for single-axis polarization.

### A minimal genetic-mechanical regulatory network that implements symmetry breaking

Provided that two radially differentiated cell types with distinct short- and long-range mechanical interactions can undergo robust, self-organized symmetry breaking (Figure 4), we next asked whether this process could be extended beyond the purely mechanical level to encompass both genetic and mechanical levels. In other words, can symmetry breaking arise from an initially homogeneous cell population in a fully self-autonomous manner, driven solely by genetic regulation and manifested through mechanical regulation as the output?

To answer this question, we built a minimal genetic–mechanical regulatory network in which each simulated cell carries a small set of genes whose activities evolve under intracellular regulation (a gene–gene interaction matrix passed through a chsigmoid/Hill nonlinearity) and intercellular regulation (a distance-decaying extracellular cue representing morphogen signaling) [Elowitz et al. *Nature* 2000; Gardner et al. Nature 2000]. Gene expression is bounded between 0 and 1 and is updated with regulation, leak, degradation, and noise. Crucially, mechanical couplings are read out from gene state: one gene-level readout modulates the short-range (adhesion/surface-tension) coefficient and a second readout modulates the directed long-range (chemotaxis-like) coupling. Thus, genes write directly into the parameters that govern cell–cell forces, enabling a homogeneous population to self-organize inside–outside heterogeneity and a single axis without pre-assigned types.

**Figure 5.**
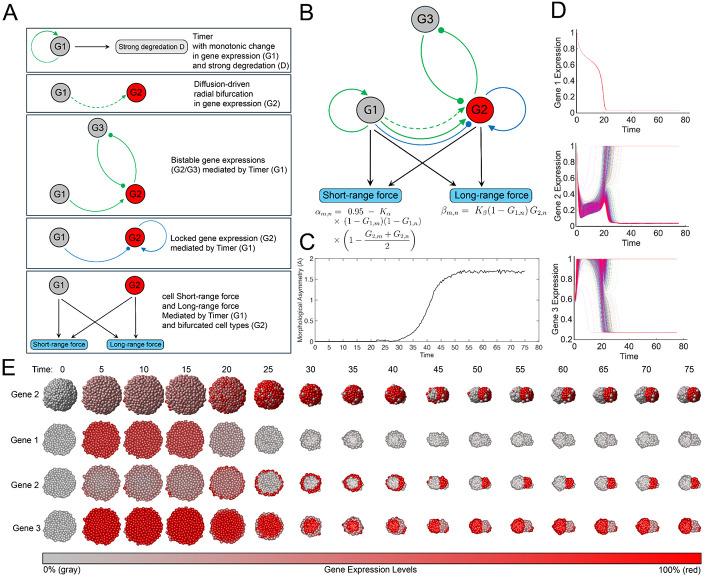
Symmetry breaking driven by differential short- and long-range forces can be implemented through a cell-autonomous genetic-mechanical regulatory network, resulting in a morphogenetic procedure in which outer cells migrate along the periphery and converge. (A) The five functional network components are dissected on the left: 1. a “*timer*” circuit with self-activation and strong degradation producing continuous monotonic change with two distinct stages; 2. diffusion-driven radial differentiation from Gene 1 to Gene 2; 3. a “*bistability*” circuit based on mutual inhibition between Gene 2 and Gene 3; 4. a “*locker*” circuit fixing high expression of Gene 2; 5. differential short- and long-range forces. (B) Schematic of an rationally designed regulatory network coupling intracellular and extracellular genetic regulation with mechanical regulation (short- and long-range forces). Short- and long-range force calculations are shown underneath. (C) Morphological asymmetry curve over *in silico* time. (D) Gene expression curves over *in silico* time. Top: monotonic dynamics of Gene 1; Middle: bifurcating dynamics of Gene 2; Bottom: bifurcating dynamics of Gene 3. Each colored trace shows the temporal gene-expression trajectory of a single simulated cell. Colors are assigned solely to distinguish cells and do not encode any additional variables. (E) Morphological evolution from a spherical aggregate to symmetry breaking governed by the genetic-mechanical regulatory network, corresponding to Movie S4. Row 1: Whole body rendered with Gene 2 expression, gray to red. Row 2: Half body (crosssection) rendered with Gene 1 expression, gray to red. Row 3: Half body (crosssection) rendered with Gene 2 expression, gray to red. Row 4: Half body (crosssection) rendered with Gene 3 expression, gray to red.

For simplicity, all gene expression levels were initialized at their maximum value (1). The design is as follows (Figure 5AB):

1. To create an “*establishment stage*” in which radially differentiated cell types are established prior to cell sorting, we implemented a globally self-activating gene, G1, with strong degradation. This generates a two-stage dynamic with an early high-expression state followed by a later low-expression state.
2. The homogeneously expressed gene, G1, activates gene G2 via extracellular stimulation, producing a pattern of higher inner expression and lower outer expression [Warmflash et al. Nat. Methods 2014].
3. An intracellular “*bistability*” circuit, active during the establishment stage but inactive during the maintenance stage, bifurcates G2 and G3 into two distinct cell types [Zhu et al. Nat. Chem. Biol. 2023; Chen et al. eLife 2025].
4. An intracellular “*locker*” circuit, inactive during establishment stage but active during maintenance stage, preserves the bifurcation of G2 and G3 into two distinct cell types [Wolpert. J. Theor. Biol. 1969].
5. Together, G1 and G2 jointly give rise to differential short- and long-range force between cells (Figure 4): G1 ensures that mechanical differentiation occurs during the maintenance stage, while G2 stabilizes the mechanical differentiation into two cell types.

Through this *de novo* design, the genetic-mechanical regulatory network fulfills the expected genetic functions and successfully reproduces symmetry breaking in a fully self-organized manner, resulting in progressively increasing morphological asymmetry (Figure 5CDE).

### *DevSim* platform allows users to explore genetic-mechanical regulatory networks and developmental dynamics efficiently.

**Figure 6.**
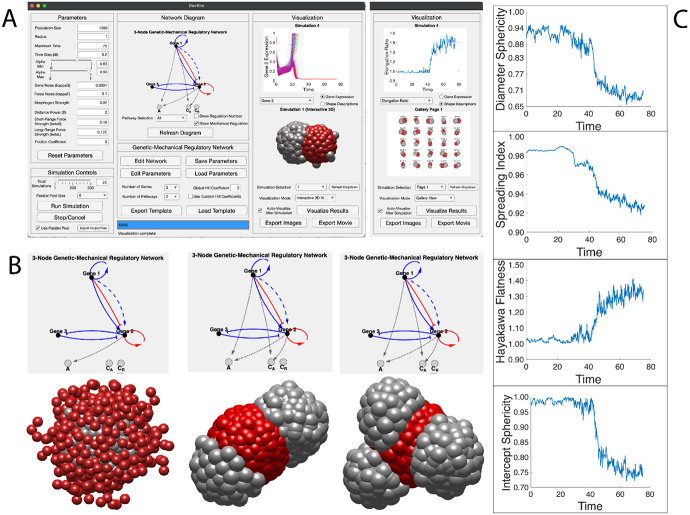
Standalone, user-friendly interface of the *DevSim* platform, accessible on both personal computers and web browsers with unique morphological developmental patterns shown. (A) The interface displays the parameters, network diagram, and developmental dynamics visualization of a genetic-mechanical regulatory network capable of robust, self-organized 3D symmetry breaking. The first visualization column illustrates gene 2 expression (top) and a 3D rendering of a bipolar symmetry-breaking pattern (bottom). The second visualization column separated by a dashed line illustrates a significant increase in shape description dynamics – elongation ratio – over time (top) and 3D renderings of the developmental patterns from 25 experimental samples in a gallery view (bottom). A detailed user guide is provided in Supplementary Text 1. (B) 3 unique patterns generated from the *DevSim* simulation framework are shown with their corresponding genetic-mechanical regulatory network diagrams shown above. Left column: 3D rendering of layered pattern morphology shown, corresponding to Movie S6. Middle column: 3D rendering of bilobed spheroid morphology shown, corresponding to Movie S7. Right column: “3D rendering of multilobed spheroid morphology shown, corresponding to Movie S8. (C) Shape description curves over time from *DevSim.* From top to bottom are described as: (1) substantial decrease of Diameter Sphericity; (2) substantial decrease of Spreading Index; (3) substantial increase of Hayakawa Flatness; (4) substantial decrease of Intercept Sphericity.

Beyond the three-node network capable of robust, self-organized 3D symmetry breaking exemplified above (Figure 5AB), the design space of potential networks is vast. It encompasses not only intracellular and intercellular genetic and mechanical regulation, but also factors such as gene number, cell number, and environmental friction, among others. To enable the exploration of customized settings, we constructed *DevSim*, a user-friendly, biophysics-based platform implemented in *Matlab*, leveraging built-in parallel computing toolbox (Figure 6A, Movie S5, Supplemental Text) [Natick, Massachusetts: The MathWorks Inc. 2024]. *DevSim* can automatically simulate the developmental dynamics of arbitrary multi-node genetic-mechanical regulatory networks across variable cell populations. The platform allows flexible adjustment of parameters, including: (1) population size and cell radius; (2) noise in gene expression and cell movement; (3) morphogen properties such as strength and spatial decay; (4) mechanical properties such as force strength and environmental friction; (5) genetic regulatory features, such as responsive concentration, expression leak, degradation rate, Hill coefficient, and configurations of series or parallel pathways; (6) mechanical regulatory features, such as adhesion and chemotaxis. It can be executed on either a personal computer (Windows/macOS) or a high-performance server, flexibly scaling from a few to thousands of processing units. Alternatively, it can be accessed through a web browser without requiring local software installation. When evaluated using the exemplary genetic-mechanical regulatory network for symmetry breaking, *DevSim* completes a full simulation — from a homogeneous spherical aggregate to a heterogeneous, elongated ellipsoid with bifurcated cell types — in less than 2 minutes on the Apple^™^ M4 Pro CPU.

In addition to the flexibility in regulatory network design, *DevSim* provides convenient tools for visualizing and analyzing developmental dynamics. The genetic-mechanical regulatory network can be automatically rendered to display an arbitrary number of genes (labeled as Gene 1, 2, 3, …), parallel pathways (with each series pathway differentially colored), regulatory edges (activation and inhibition), and regulatory types (solid lines for intracellular regulation and dashed lines for intercellular regulation) (Figure 6B). Besides, *DevSim* also outputs per-cell trajectories of gene expression and whole-body shape descriptors over time. To characterize the overall shape, twelve 3D shape descriptors adapted from our previous work [Guan et al. Membranes 2024] are included: General Sphericity, Diameter Sphericity, Intercept Sphericity, Maximum Projection Sphericity, Hayakawa Roundness, Spreading Index, Elongation Ratio, Pivotability Index, Wilson Flatness Index, Hayakawa Flatness Ratio, Huang Shape Factor, and Corey Shape Factor (Figure 6C, Figure S12, Table S2). Furthermore, *DevSim* can generate and rotate 3D patterns of cell positions and gene expressions, with outputs available as images or movies. As demonstrations, *DevSim* successfully simulates the symmetry breaking driven by a monotonic timer and cell-type bifurcation, with both gene expression and shape description displayed (Figure 6A). Also, alternative network topology and parameters producing self-organized layered patterns as well as bilobed-to-multilobed patterns are also visualized (Figure 6B, Movies S6-S8).

## DISCUSSION

Development involves diverse forms of pattern formation across scales, ultimately giving rise to functional tissues, organs, and entire organisms. Among these patterns, symmetry breaking is especially critical, as it establishes the axes of the body and of individual tissues or organs, driving both morphological and genetic differentiation. In this study, we focused on the remarkable self-organizing capacity for symmetry breaking observed in mammalian gastruloid systems (Figure 1) and sought to address a fundamental question: *which cell-cell interactions can robustly generate this outcome?* By systematically enumerating short-range interactions, such as adhesion between two radially differentiated cell types, we found that classic adhesion-driven sorting mechanisms alone are not sufficiently robust. In contrast, a complete morphogenetic landscape analysis revealed that incorporating long-range attraction among outer cells, in combination with differential adhesion, represents the most efficient and minimal design principle for robust symmetry breaking. To enable broader investigations of developmental pattern formation, we constructed the *DevSim* platform, a flexible framework capable of simulating arbitrary genetic–mechanical regulatory networks and generating outputs ranging from symmetry breaking to a wide spectrum of developmental patterns.

While exhaustive theoretical analyses highlight the significant role of long-range attraction in uniting dispersed cells, it remains unclear whether such interactions actually occur in biological systems or what molecular mechanisms might underlie them. A leading candidate is chemotaxis, where cells secrete diffusible molecules (morphogens) that establish gradients, which neighboring cells can detect and respond to through polarization or frequent directional adjustments. To probe whether such mechanisms operate in mammalian gastruloids, several experimental strategies can be employed: (1) Adhesive protein elimination: Dissociate mammalian gastruloids by disrupting adhesive proteins while keeping cells viable, and then monitor their spatial interactions or movements relative to each other or the gastruloid [Mayran et al. bioRxiv 2023]. (2) Whole-gastruloid tracking: Apply 3D time-lapse imaging of gastruloids at cellular resolution to assess whether cell movements are directional and coherent [Gros et al. eLife 2025]. (3) Inter-gastruloid influence: Culture two or more gastruloids in close proximity to test whether their mutual influence alters cell movements or expected symmetry-breaking behaviors [Anlaş et al. bioRxiv 2021; Anand et al. Cell 2023]. Finally, both intra- and extra-gastruloid cell movements can be further interrogated by perturbing diffusive signaling pathways, including but not limited to BMP, Wnt, Nodal, FGF [Liu et al. *Stem Cell Rep.* 2021; Gattiglio et al. Biol. Open 2023]. These newly identified long-range mechanical interactions could serve as novel bioparts for synthetic biology, expanding the currently limited repertoire of cell-adhesion mechanisms.

Regulatory mechanisms can also be explored computationally. Using the *DevSim* platform, it is in principle possible to identify genetic-mechanical regulatory networks capable of driving cell-autonomous, self-organized pattern formation. Although the design space is vast — with the number of parameters increasing combinatorially with gene count and regulatory edge combinations — various optimization strategies can be employed, including gradient descent, genetic algorithms, and artificial bee colony algorithms. A particularly powerful alternative is automatic differentiation, which records all time-iteration dynamics and backpropagates information from terminal pattern outcomes to the initial parameter space [Baydin et al. J. Mach. Learn. 2017; Mottes et al. Artificial Life Conference 2023]. This approach enables efficient identification of both network topologies and parameter sets. The resulting feasible network designs can reveal key biological insights, such as distinctive gene dynamics, regulatory motifs, and critical genetic or mechanical features — including degradation rates, response sensitivities, and specific versus non-specific adhesion. These predictions can then be cross-referenced with established biological knowledge — for example, the known mutual inhibition between Wnt and Nodal signaling and associated differential cell adhesion [McNamara et al. Nat. Cell Biol. 2024; Dias et al. bioRxiv 2025] — and may further uncover previously unknown or underappreciated regulatory logics.

In the future, investigations of design principles can be extended beyond symmetry breaking, as discussed in this study, to other forms of pattern formation. Examples include periodic structures and multi-node arrangements that mimic the heterogeneous cell type distribution within pancreatic islets. Such exploration can be achieved by customizing initial conditions — such as cell-type composition, spatial distribution, and population size — as well as incorporating diverse shape descriptors. A rich collection of large-scale open-resource datasets on natural developmental patterns — spanning worm (*ITK-SNAP-CVE* and *CMOS*) [Guan et al. Nat. Commun. 2025], ascidian (*MorphoSeq*) [Sladitschek et al. Cell 2020], fruit fly (*Flysta3D-v2*) [Wang et al. Cell 2025], zebrafish (*Zebrahub*) [Lange et al. Cell 2024], mouse (http://most.ccla.ac.cn) [Qu et al. Nat. Commun. 2023], and human (http://cs8.3dembryo.com) [Xiao et al. Cell 2024] systems — could be systematically analyzed using *DevSim*, an open and practical framework for multicellular modeling and hypothesis testing. *DevSim* provides users with flexible control over genetic–mechanical rule sets, efficient parameter management, and multiple readout options to capture emergent developmental dynamics and multicellular patterning. Beyond natural systems, synthetic models aimed at uncovering developmental principles and advancing tissue or organ engineering could also benefit. By leveraging biophysical guidance for self-organized pattern formation, these approaches can reduce or even eliminate the manual effort required to aggregate cells or chemically manipulate their states, thereby streamlining synthetic developmental design.

While the current computational frameworks that integrate mechanical and genetic regulation are useful for dissecting rules of developmental pattern formation, addressing the roles and details of additional biophysical processes will require a comprehensive model upgrade. (1) In the coarse-grained model, the balance distance between neighboring cells (*i.e.*, $|d_{m,n(b)}|=\alpha_{m,n}(l_{m}+l_{n})$) can approximate the joint effects of adhesion and its opposing force surface tension, considering cell-cell distance. However, the broader consequences of surface tension (*e.g.*, the curvature and area of cell-cell contact interfaces), which influence intercellular forces as well as the secretion and reception of signaling molecules, require higher-dimensional representations [Kajita et al. Bioinformatics 2003; Kuang et al. PLoS Comput. Biol. 2022]. (2) The chemotaxis-like movement is modeled as a straightforward pairwise instantaneous cell-to-cell force, neglecting microscopic details such as how gradients of morphogens and other substances are established in space and time [Yaman et al. *Cell 2023*; Al-Hilal et al. Nat. Cell Biol. 2025], as well as cellular mechanisms like polarity that controls directional movement [Nielsen et al. iScience 2020; Cao et al. eLife 2019], or concentration sensing, as exemplified by the run-and-tumble motion of bacteria [Hu et al. *PLoS Comput. Biol.* 2014; Nakamura et al. *Phys. Rev. Res.* 2022]. (3) Although the cell number was fixed to match that of a 72-h gastruloid, developing biological systems typically undergo continuous cell divisions, and experiments have shown that the self-organizing capacity for symmetry breaking depends on gastruloid size [Fiuza et al. Cells Dev. 2025; Bennabi et al. Sci. Adv. 2025]. A more realistic investigation should therefore incorporate cell division, which could be implemented by splitting one cell into two either randomly or according to a defined cell cycle as executed before [Nissen et al. eLife 2018; Tian et al. Phys. Biol. 2020]. Future extensions of our model that incorporate additional mechanisms, together with quantitative matching of numbers and units to experimental systems, will help clarify the design principles of developmental patterning and reconcile theory with observation.

## STAR★METHODS

Detailed methods are provided in the online version of this paper and include the following:

### RESOURCE AVAILABILITY

#### Lead Contact

Further information and requests for resources and reagents should be directed to and will be fulfilled by the Lead Contact, Sahand Hormoz (sahand_hormoz@hms.harvard.edu).

#### Materials availability

This study did not generate new unique reagents.

#### Data availability

The data will be available upon publication.

#### Code availability

The code will be available at https://github.com/hormoz-lab.

### EXPERIMENTAL MODEL AND SUBJECT DETAILS

Human ES cells were maintained in NutriStem hPSC XF medium (Sartorius, 05-100-1B) on Vitronectin-coated plates (VTN-N; Thermo Fisher Scientific, A14700). To prepare cells for generating gastruloids, 3x10^4^ RUES2-GLR hES cells [Martyn et al. Nature 2018] were seeded onto Vitronectin-coated 6-well multi-well plates in Nutristem and supplemented with 10 μM Y-27632 dihydrochloride (ROCK inhibitor; Tocris) for the first 24h after seeding. Media changes were performed daily. On day 4, cells were pre-treated with 3.25 μM Chir (Tocris). On day 5, cells were harvested and dissociated into single cells. 400 cells were transferred into 96-well U-bottom multi-well plates in 40 μl E6 media supplemented with ROCKi and 0.5 μM Chir. After 24h, 150 μl per well E6 media was added. E6 media was refreshed subsequently every 24h by removing 150 μl media and adding 150 μl fresh E6 media.

### METHOD DETAILS

#### Confocal Fluorescence microscopy

Live imaging of human gastruloids was performed on a Yokogawa CSU-W1 spinning-disk confocal mounted on a Nikon Ti2 motorized inverted microscope equipped with Perfect Focus System (PFS), controlled by NIS-Elements (Nikon). The system includes a Nikon LUN-F XL solid-state laser combiner (405/445/488/514/561/640 nm), a Hamamatsu ORCA-Fusion BT sCMOS camera, and an Okolab stage-top incubator for on-stage environmental control. All imaging was done using a 20×/0.75 Plan Apo DIC objective.

For fluorescence imaging, we imaged RUES2-GLR hESC-derived gastruloids (containing fluorescent reporters as follows: SOX17-tdTomato, BRA/TBXT-mCerulean, SOX2-mCitrine). Using the following laser and Chroma (ET) filter sets: mCerulean (445 nm laser; emission 480/40), mCitrine (514 nm laser; emission 535/30), and tdTomato (561 nm laser; emission 620/60). Acquisition proceeded in the order 445→514→561 to minimize cross-excitation and photobleaching. For time-lapse experiments, gastruloids were maintained at 37 °C, 5% CO_2_, and high humidity in the Okolab enclosure. Laser power and exposure times were adjusted to the minimum required to achieve adequate SNR while avoiding phototoxicity.

#### Brightfield microscopy

Live imaging of human gastruloids was performed on a Nikon Ti2 motorized inverted microscope equipped with Perfect Focus System (PFS), controlled by NIS-Elements (Nikon). The system includes a Hamamatsu Flash4.0 sCMOS camera (6.5 μm^2^ photodiode), Nikon motorized stage and emission filter wheel, and an Okolab stage-top incubator for on-stage environmental control. All imaging was done using a 10x/0.45 Plan Apo λ objective.

For time-lapse experiments, we imaged H9 SOX17mCHERRY/w;RUNX1CGFP/w hESC-derived gastruloids. Gastruloids were maintained at 37 °C, 5% CO_2_, and high humidity in the Okolab enclosure.

#### Biophysical model

Biophysical model is detailed in the attached document, Biophysical model.pdf.

#### Platform construction

The *DevSim* (Development Simulator) platform was constructed for simulating genetic-mechanical regulatory networks and developmental dynamics, as a numerical implementation following exactly the descriptions in METHOD DETAILS - Biophysical Model. It is based on MATLAB R2024b [Natick, Massachusetts: The MathWorks Inc. 2024], leveraging MATLAB App Designer for the user interface and Parallel Computing Toolbox for efficient large-scale simulations. The platform provides a fully executable framework for the symmetry-breaking mechanisms reported in this study. After extensive testing, it has been customized to run seamlessly on personal computers (Windows 11 and macOS Sequoia 15.6.1), online webpage (https://www.mathworks.com/products/matlab-online), and high-performance servers (O2 High Performance Compute Cluster at Harvard Medical School, https://harvardmed.atlassian.net/wiki/spaces/O2). A complete user guidebook is provided in the Supplemental Text.

### QUANTITATIVE AND STATISTICAL ANALYSIS

#### Developmental Pattern Description

In this study, we quantify developmental patterns composed of two cell types, $t_{1}$ with $N_{t_{1}}$ cells and $t_{2}$ with $N_{t_{2}}$ cells, using two metrics: cell loss (L) and morphological asymmetry (A).

For computational simulations, the largest connected aggregate is first identified, defined as the group of cells in which each cell is within a distance less than 2l from at least one neighbor. The numbers of $t_{1}$ and $t_{2}$ cells in this aggregate are denoted ${N_{t_{1}}}^{’}$ and ${N_{t_{2}}}^{’}t_{2}$ respectively. Cell loss is then calculated as:

$$L=\frac{\left(N_{t_{1}}+N_{t_{2}}\right)-\left({N_{t_{1}}}^{’}+{N_{t_{2}}}^{’}\right)}{\left(N_{t_{1}}+N_{t_{2}}\right)}$$

For the same aggregate, let $r_{t_{1},i}$ and $r_{t_{2},i}$ represent the positions of the i-th cell of type $t_{1}$ and $t_{2}$ respectively. The centroid distance between the two cell types is given by:

$$D_{t_{1},t_{2}}=|\frac{1}{{N_{t_{1}}}^{’}}\sum_{i\in t_{1}}r_{t_{1},i}-\frac{1}{{N_{t_{2}}}^{’}}\sum_{i\in t_{2}}r_{t_{2},i}|$$

The average distance to the overall centroid (considering all ${N_{t_{1}}}^{’}+{N_{t_{2}}}^{’}$ cells) is given by:

$$\bar{D}=\frac{1}{{N_{t_{1}}}^{’}+{N_{t_{2}}}^{’}}|\sum_{i\in t_{1}\cup t_{2}}\left(r_{i}-\frac{1}{{N_{t_{i}}}^{’}+{N_{t_{2}}}^{’}}\sum_{i\in t_{1}\;or\;t_{2}}r_{i}\right)|$$

Finally, morphological asymmetry is then calculated as:

$$A=\frac{D_{t_{1},t_{2}}}{\bar{D}}\cdot \frac{{N_{t_{1}}}^{’}}{N_{t_{1}}}\cdot \frac{{N_{t_{2}}}^{’}}{N_{t_{2}}}$$

## Supplementary Material

Supplement 1

Supplement 2

Supplement 3

Supplement 4

Supplement 5

Supplement 6

Supplement 7

Supplement 8

Supplement 9

Supplement 10

Supplement 11

Supplement 12

Supplemental information can be found along this paper.

## Figures and Tables

**Table:** • KEY RESOURCES TABLE

REAGENT or RESOURCE	SOURCE	IDENTIFIER
**Reagents**		
NutriStem hPSC XF Medium	Sartorius	Catalog # 05-100-1B
Vitronectin (VTN-N)	Thermo Scientific	Catalog # A14700
Essential 6 (E6) Medium	Thermo Scientific	Catalog # A1516401
96W Cell-Repellent Plate, Round (U) Bottom	Greiner Bio-One	Catalog # 650970
Y-27632 dihydrochloride	Tocris	Catalog # 1254
CHIR99021	Tocris	Catalog # 4423
**Cell lines**		
RUES2-GLR	[Martyn et al. Nature 2018]	RUESe002-A-6 (RRID:CVCL_E9CV)
H9 SOX17^mCHERRY/w^;RUNX1C^GFP/w^	[Ng et al. Nat. Biotechnol. 2016]	Parental cell line: H9 (WA09), (RRID: CVCL_9773)
