# Supplementary material for: Cooperative short- and long-range interactions enable robust symmetry breaking and axis formation
Source: bioRxiv. 2025 Sep 29:2025.09.27.678924. Preprint. [Version 1] doi: 10.1101/2025.09.27.678924 (PMC12621816; doi:10.1101/2025.09.27.678924)
Supplement: Supplement 11 [file media-11.pdf]

Cooperative short- and long-range interactions enable robust symmetry breaking and axis formation

Guoye Guan (关国业)[1,2†], Suxuan Wang (王苏轩)[1,2†], T. Glenn Shields (习格义)[3,4†],
Seong Ho Pahng[1,2,5], Claire Xinyu Shao[1,2], Juns Ye (叶俊仕)[2,6], Christoph Budjan[1,2*], Sahand Hormoz[1,2,7,8*]

1 Department of Systems Biology, Harvard Medical School, Boston, MA 02115, USA

2 Department of Data Science, Dana-Farber Cancer Institute, Boston, MA 02215, USA

3 Harvard College, Faculty of Arts and Sciences, Harvard University, Boston, MA 02138, USA

4 Department of Molecular and Cellular Biology, Harvard University, Boston, MA 02138, USA

5 Department of Chemistry and Chemical Biology, Harvard University, Boston, MA 02138, USA

6 Boston Latin School, Boston, MA 02115, USA

7 Broad Institute of MIT and Harvard, Boston, MA 02142, USA

8 Lead Contact

† These authors contributed equally to this work

** Correspondence: cbudjan@hms.harvard.edu (C.B.); sahand_hormoz@hms.harvard.edu (S.H.)*

Supplemental Figure

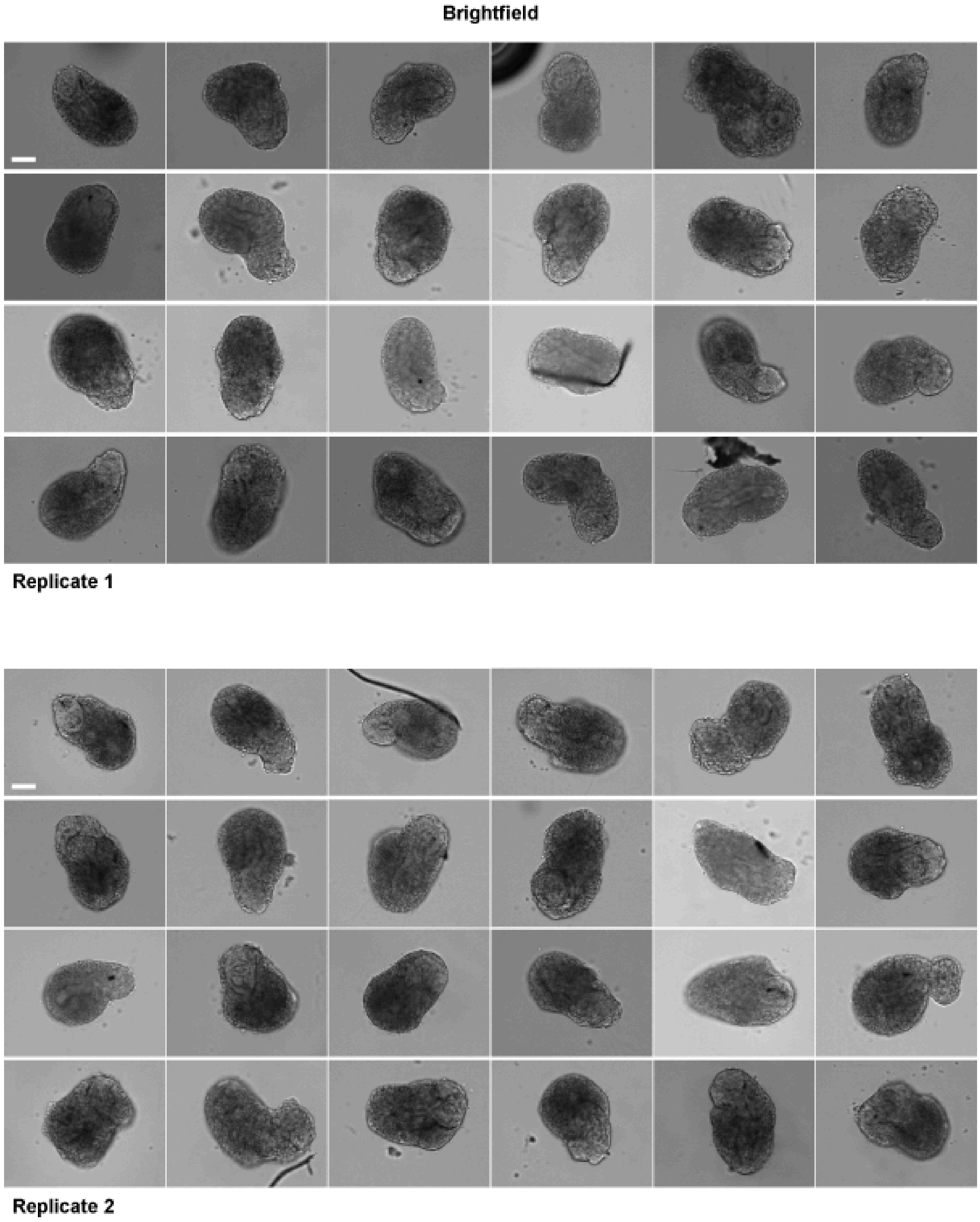

Figure S1. Human gastruloid exhibit robust and reproducible self-organized symmetry breaking.

Brightfield images of two independent experimental replicates of RUES2-GLR hES-derived human gastruloids (24 gastruloids each) 72 hours post-seeding. Scale bar: 100 µm.

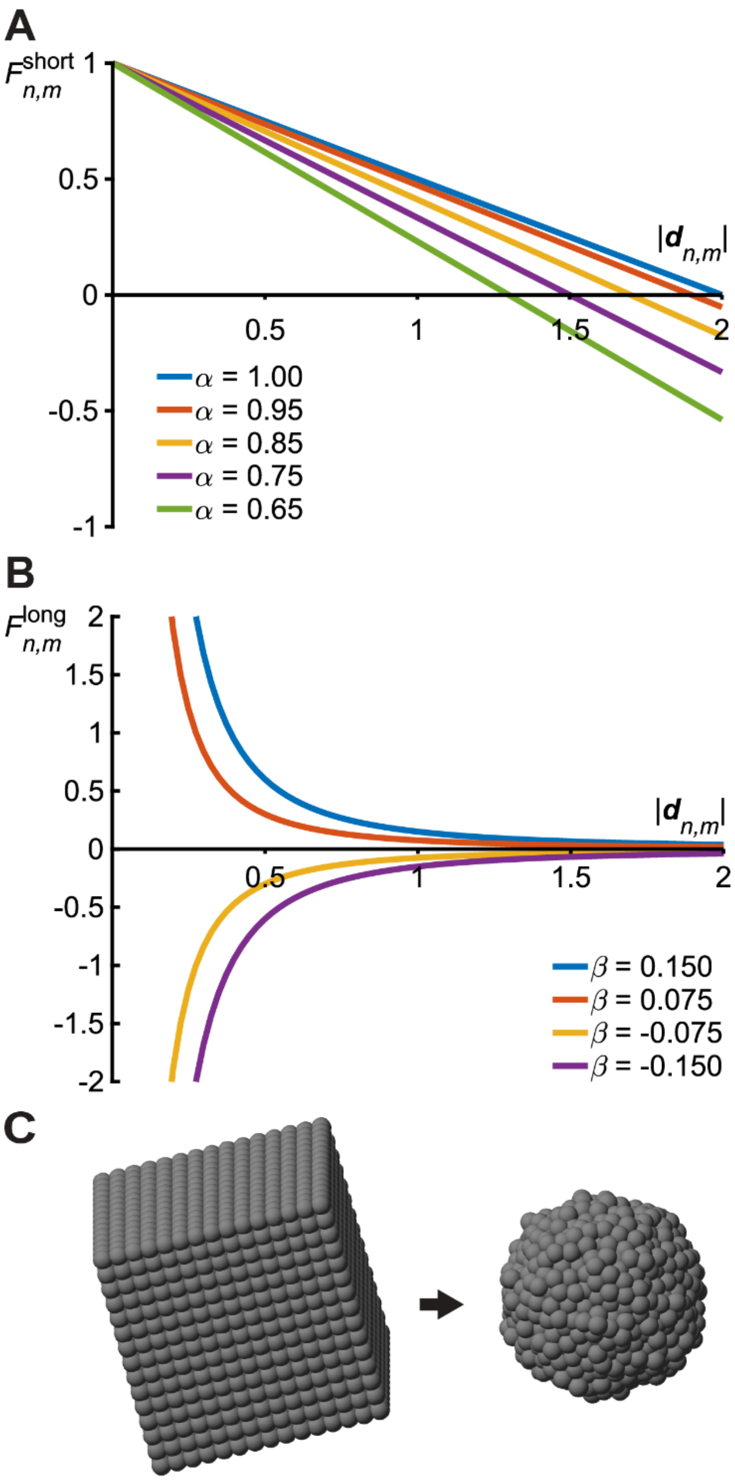

Figure S2. Details of mechanical model and simulation.

(A) Short-range force curves as a function of distance between cell m and cell n, shown for α values of 0.65, 0.75, 0.85, 0.95, and 1.0.

(B) Long-range force curves as a function of distance between cell m and cell n, shown for β values of -0.150, -0.075, 0.075, 0.150.

(C) Simulation (time step length $\Delta T = 0.01$; total time duration $T_{total} = 100$) for randomizing initial positions of a cube of 15×15×15 regularly arranged cells, with homogeneous short-range force ($\alpha = 0.80$), retaining the 1,500 cells closest to the overall centroid in the end.

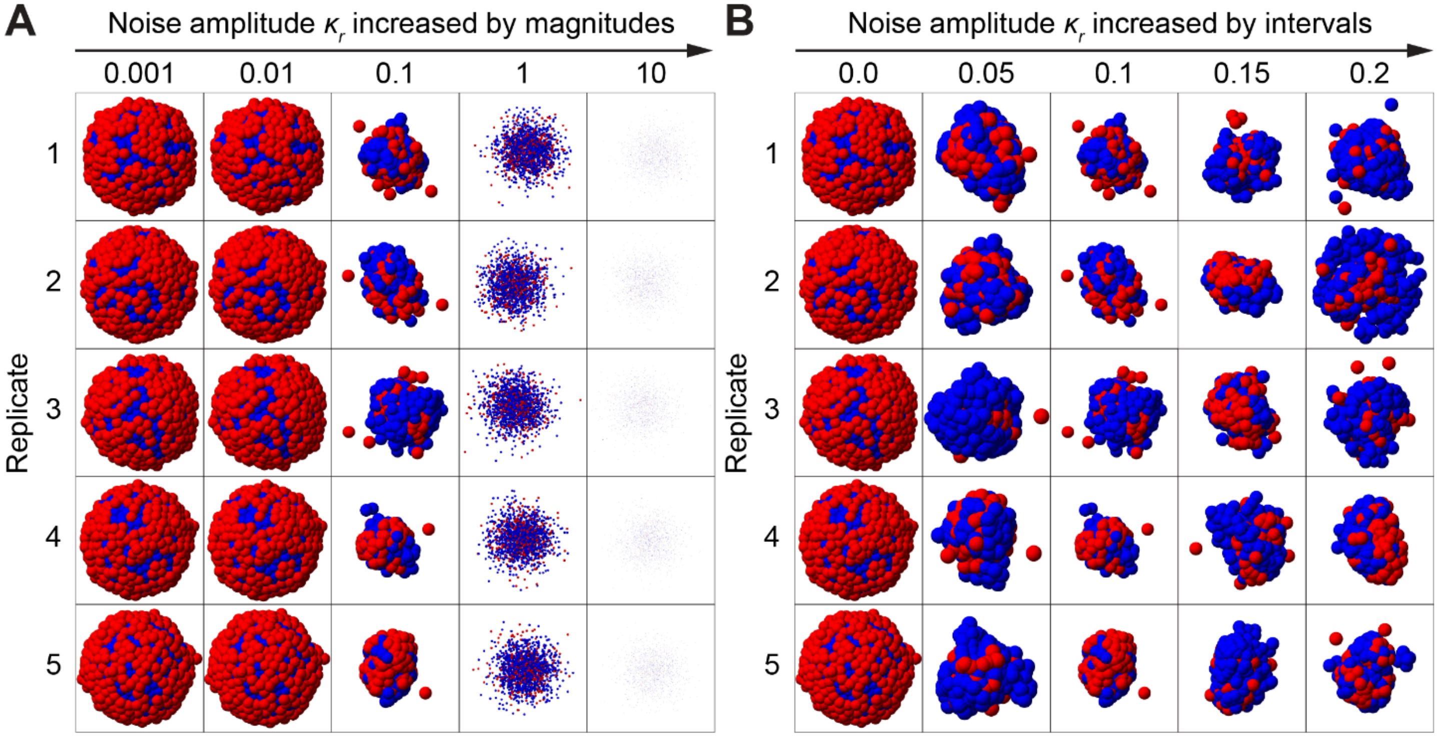

Figure S3. Final morphologies evolved from a spherical aggregate under different noise levels, when $\left(\alpha_{i-i}, \alpha_{o-o}, \alpha_{i-o}\right) = (0.750, 0.725, 0.800)$.

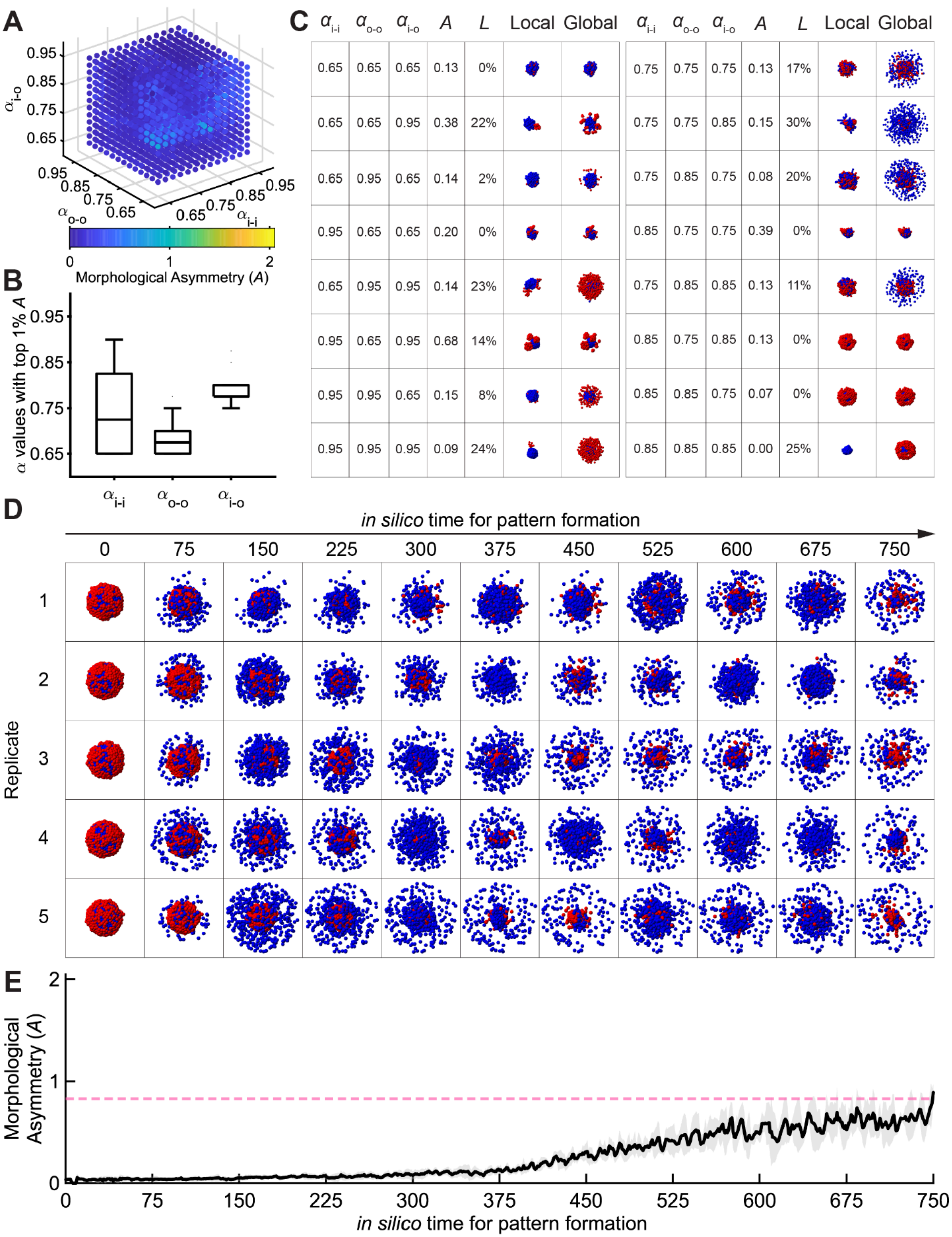

A
α i-o
0.95
0.85
0.75
0.65
α o-o
α i-i
Morphological Asymmetry (A)
0
1
2
B
α values with top 1% A
α i-i
α o-o
α i-o
C
α i-i | α o-o | α i-o | A | L | Local | Global
0.65 | 0.65 | 0.65 | 0.13 | 0%
0.65 | 0.65 | 0.95 | 0.38 | 22%
0.65 | 0.95 | 0.65 | 0.14 | 2%
0.95 | 0.65 | 0.65 | 0.20 | 0%
0.65 | 0.95 | 0.95 | 0.14 | 23%
0.95 | 0.65 | 0.95 | 0.68 | 14%
0.95 | 0.95 | 0.65 | 0.15 | 8%
0.95 | 0.95 | 0.95 | 0.09 | 24%
0.75 | 0.75 | 0.75 | 0.13 | 17%
0.75 | 0.75 | 0.85 | 0.15 | 30%
0.75 | 0.85 | 0.75 | 0.08 | 20%
0.85 | 0.75 | 0.75 | 0.39 | 0%
0.75 | 0.85 | 0.85 | 0.13 | 11%
0.85 | 0.75 | 0.85 | 0.13 | 0%
0.85 | 0.85 | 0.75 | 0.07 | 0%
0.85 | 0.85 | 0.85 | 0.00 | 25%
D
in silico time for pattern formation
0 75 150 225 300 375 450 525 600 675 750
Replicate
1
2
3
4
5
E
Morphological Asymmetry (A)
in silico time for pattern formation

Figure S4. Morphogenetic landscape consisting of adhesion $\left(\alpha_{i-i}, \alpha_{o-o}, \alpha_{i-o}\right)$ and long-range attraction $\beta_{i\to i} = 0.15$.

(A) Heatmap showing morphological asymmetry (A) across the three short-range force parameters $\left(\alpha_{i-i}, \alpha_{o-o}, \alpha_{i-o}\right)$ (blue: low A; yellow: high A), revealing maximum $A = 0.891$.

(B) Boxplot showing the α value distribution for parameter combinations within the top 1% of A.

(C) Representative final morphologies evolved from a spherical aggregate, when the three short-range force parameters $\left(\alpha_{i-i}, \alpha_{o-o}, \alpha_{i-o}\right)$ are set as regular values. Results with extreme α values (0.65, mimicking strong adhesion; 0.95, mimicking weak adhesion) are shown on the left; results with moderate α values (0.75 and 0.85, mimicking moderate adhesion) are shown on the right. Here, the local pattern shows the largest aggregate with the most contacting cells; the global pattern shows the entirety of cells within the simulated system.

(D) Morphological evolution from a spherical aggregate to weak or no symmetry breaking. Shown are five independent replicates with the highest morphological asymmetry observed under adhesion parameters $\left(\alpha_{i-i}, \alpha_{o-o}, \alpha_{i-o}\right) = (0.800, 0.750, 0.800)$.

(E) Morphological asymmetry curve over *in silico* time, plotted from the five independent replicates with the highest morphological asymmetry observed under short-range force parameters $\left(\alpha_{i-i}, \alpha_{o-o}, \alpha_{i-o}\right) = (0.800, 0.750, 0.800)$. Black solid line: mean; gray shade: standard deviation; pink dashed line: the maximum final A value when $\beta = 0$, corresponding to Figure 3.

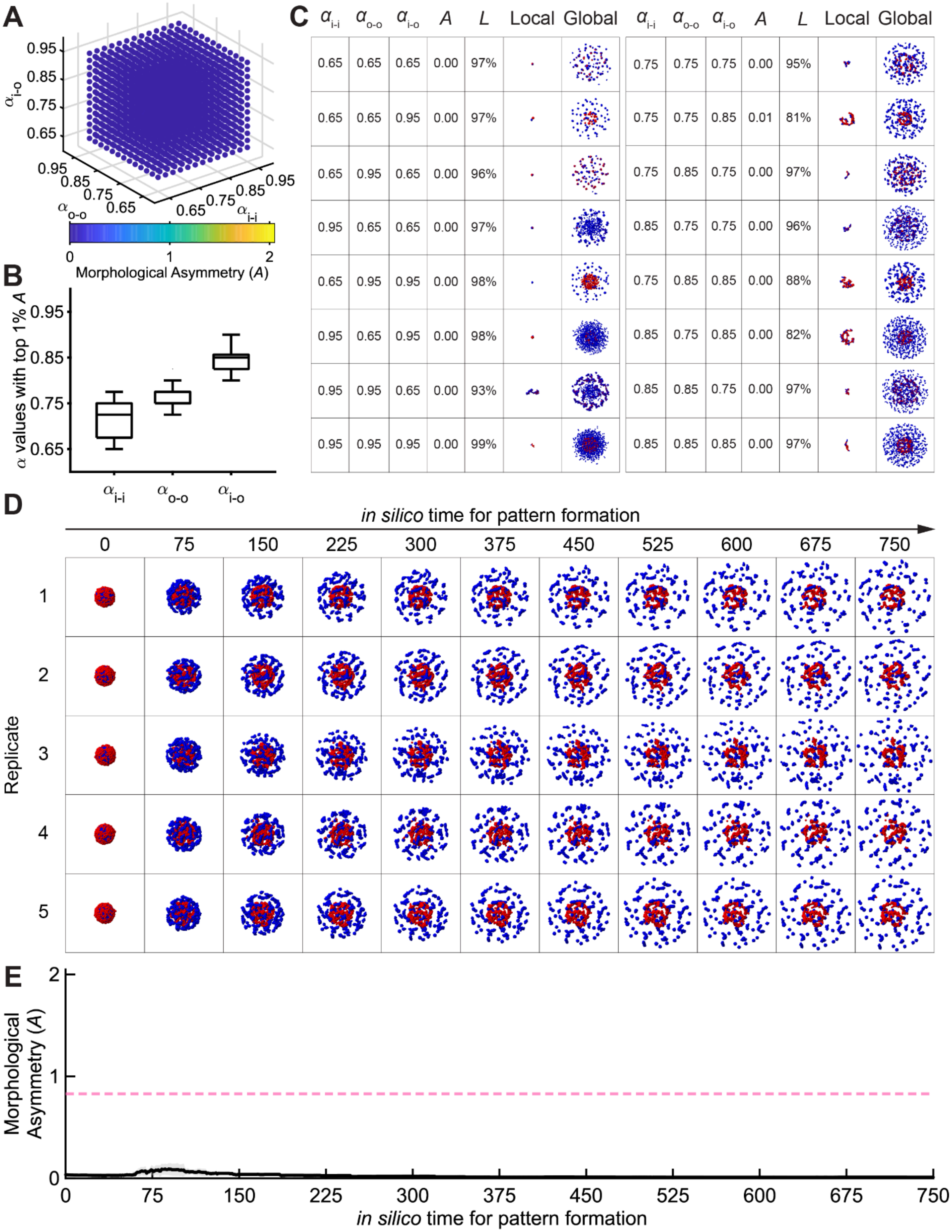

A
α_{i-o}
0.95
0.85
0.75
0.65
α_{o-o}
α_{i-i}
Morphological Asymmetry (A)
0
1
2
B
α values with top 1% A
α_{i-i}
α_{o-o}
α_{i-o}
C
α_{i-i} α_{o-o} α_{i-o} A L Local Global
0.65 0.65 0.65 0.00 97%
0.65 0.65 0.95 0.00 97%
0.65 0.95 0.65 0.00 96%
0.95 0.65 0.65 0.00 97%
0.65 0.95 0.95 0.00 98%
0.95 0.65 0.95 0.00 98%
0.95 0.95 0.65 0.00 93%
0.95 0.95 0.95 0.00 99%
0.75 0.75 0.75 0.00 95%
0.75 0.75 0.85 0.01 81%
0.75 0.85 0.75 0.00 97%
0.85 0.75 0.75 0.00 96%
0.75 0.85 0.85 0.00 88%
0.85 0.75 0.85 0.00 82%
0.85 0.85 0.75 0.00 97%
0.85 0.85 0.85 0.00 97%
D
in silico time for pattern formation
0 75 150 225 300 375 450 525 600 675 750
Replicate
1
2
3
4
5
E
Morphological Asymmetry (A)
2
1
0
0 75 150 225 300 375 450 525 600 675 750
in silico time for pattern formation

Figure S5. Morphogenetic landscape consisting of adhesion $\left(\alpha_{i-i}, \alpha_{o-o}, \alpha_{i-o}\right)$ and long-range attraction $\beta_{i\rightarrow i} =- 0.15$.

(A) Heatmap showing morphological asymmetry (A) across the three short-range force parameters $\left(\alpha_{i-i}, \alpha_{o-o}, \alpha_{i-o}\right)$ (blue: low A; yellow: high A), revealing maximum $A = 0.010$.

(B) Boxplot showing the α value distribution for parameter combinations within the top 1% of A.

(C) Representative final morphologies evolved from a spherical aggregate, when the three short-range force parameters $\left(\alpha_{i-i}, \alpha_{o-o}, \alpha_{i-o}\right)$ are set as regular values. Results with extreme α values (0.65, mimicking strong adhesion; 0.95, mimicking weak adhesion) are shown on the left; results with moderate α values (0.75 and 0.85, mimicking moderate adhesion) are shown on the right. Here, the local pattern shows the largest aggregate with the most contacting cells; the global pattern shows the entirety of cells within the simulated system.

(D) Morphological evolution from a spherical aggregate to weak or no symmetry breaking. Shown are five independent replicates with the highest morphological asymmetry observed under adhesion parameters $\left(\alpha_{i-i}, \alpha_{o-o}, \alpha_{i-o}\right) = (0.675, 0.750, 0.825)$.

(E) Morphological asymmetry curve over *in silico* time, plotted from the five independent replicates with the highest morphological asymmetry observed under short-range force parameters $\left(\alpha_{i-i}, \alpha_{o-o}, \alpha_{i-o}\right) = (0.675, 0.750, 0.825)$. Black solid line: mean; gray shade: standard deviation; pink dashed line: the maximum final A value when $\beta = 0$, corresponding to Figure 3.

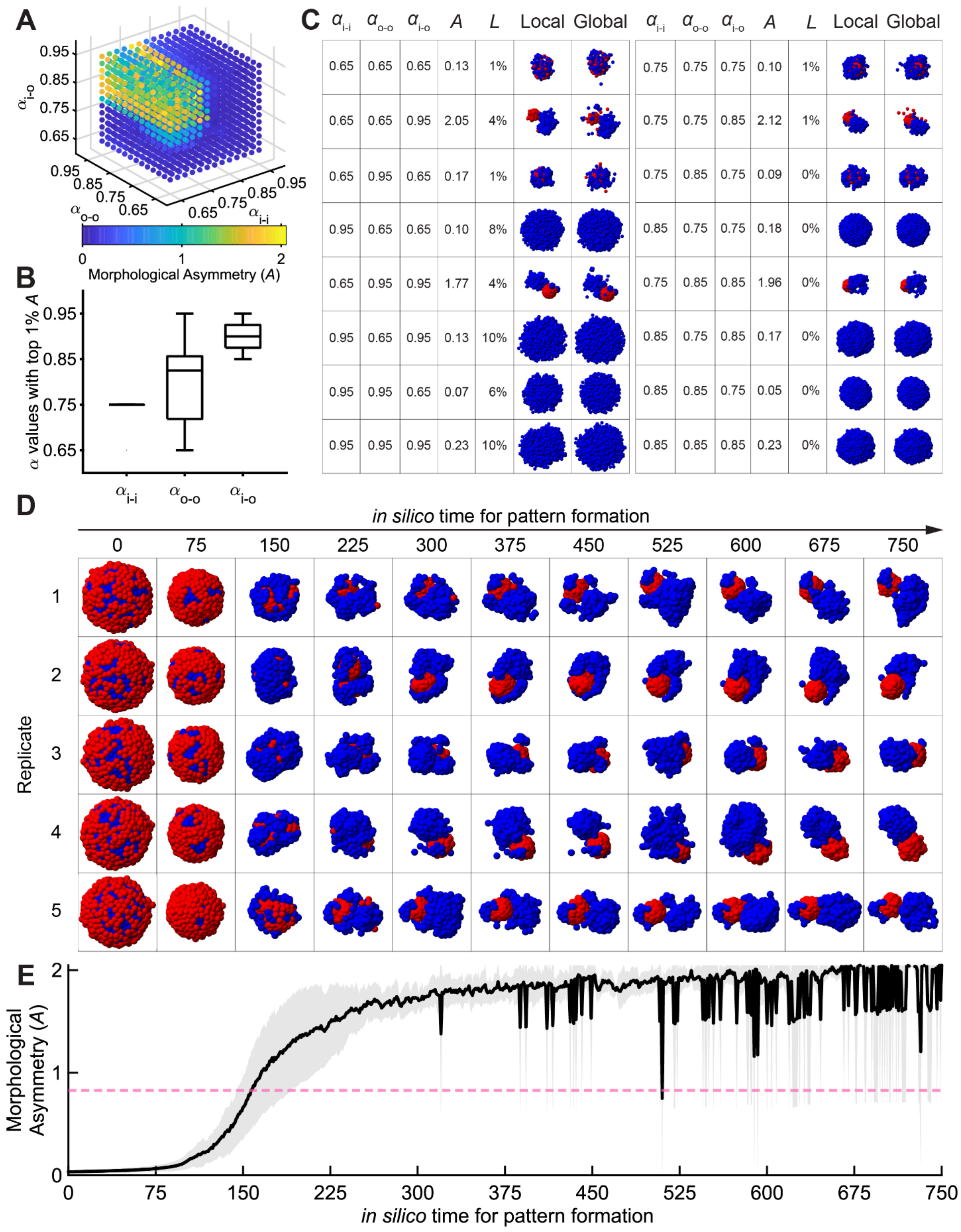

A
0.95
0.85
0.75
0.65
α_{i-o}
α_{o-o}
α_{i-i}
0 1 2
Morphological Asymmetry (A)
B
α values with top 1% A
0.95
0.85
0.75
0.65
α_{i-i} α_{o-o} α_{i-o}
C
α_{i-i} α_{o-o} α_{i-o} A L Local Global
0.65 0.65 0.65 0.13 1%
0.65 0.65 0.95 2.05 4%
0.65 0.95 0.65 0.17 1%
0.95 0.65 0.65 0.10 8%
0.65 0.95 0.95 1.77 4%
0.95 0.65 0.95 0.13 10%
0.95 0.95 0.65 0.07 6%
0.95 0.95 0.95 0.23 10%
α_{i-i} α_{o-o} α_{i-o} A L Local Global
0.75 0.75 0.75 0.10 1%
0.75 0.75 0.85 2.12 1%
0.75 0.85 0.75 0.09 0%
0.85 0.75 0.75 0.18 0%
0.75 0.85 0.85 1.96 0%
0.85 0.75 0.85 0.17 0%
0.85 0.85 0.75 0.05 0%
0.85 0.85 0.85 0.23 0%
D
in silico time for pattern formation
0 75 150 225 300 375 450 525 600 675 750
Replicate
1
2
3
4
5
E
Morphological Asymmetry (A)
0 1 2
0 75 150 225 300 375 450 525 600 675 750
in silico time for pattern formation

Figure S6. Morphogenetic landscape consisting of adhesion $\left(\alpha_{i-i}, \alpha_{o-o}, \alpha_{i-o}\right)$ and long-range attraction $\beta_{o\rightarrow o} = 0.15$

.

(A) Heatmap showing morphological asymmetry (A) across the three short-range force parameters $\left(\alpha_{i-i}, \alpha_{o-o}, \alpha_{i-o}\right)$ (blue: low A; yellow: high A), revealing maximum $A = 2.047$.

(B) Boxplot showing the α value distribution for parameter combinations within the top 1% of A.

(C) Representative final morphologies evolved from a spherical aggregate, when the three short-range force parameters $\left(\alpha_{i-i}, \alpha_{o-o}, \alpha_{i-o}\right)$ are set as regular values. Results with extreme α values (0.65, mimicking strong adhesion; 0.95, mimicking weak adhesion) are shown on the left; results with moderate α values (0.75 and 0.85, mimicking moderate adhesion) are shown on the right. Here, the local pattern shows the largest aggregate with the most contacting cells; the global pattern shows the entirety of cells within the simulated system.

(D) Morphological evolution from a spherical aggregate to weak or no symmetry breaking. Shown are five independent replicates with the highest morphological asymmetry observed under adhesion parameters $\left(\alpha_{i-i}, \alpha_{o-o}, \alpha_{i-o}\right) = (0.750, 0.825, 0.950)$.

(E) Morphological asymmetry curve over *in silico* time, plotted from the five independent replicates with the highest morphological asymmetry observed under short-range force parameters $\left(\alpha_{i-i}, \alpha_{o-o}, \alpha_{i-o}\right) = (0.750, 0.825, 0.950)$. Black solid line: mean; gray shade: standard deviation; pink dashed line: the maximum final A value when $\beta = 0$, corresponding to Figure 3.

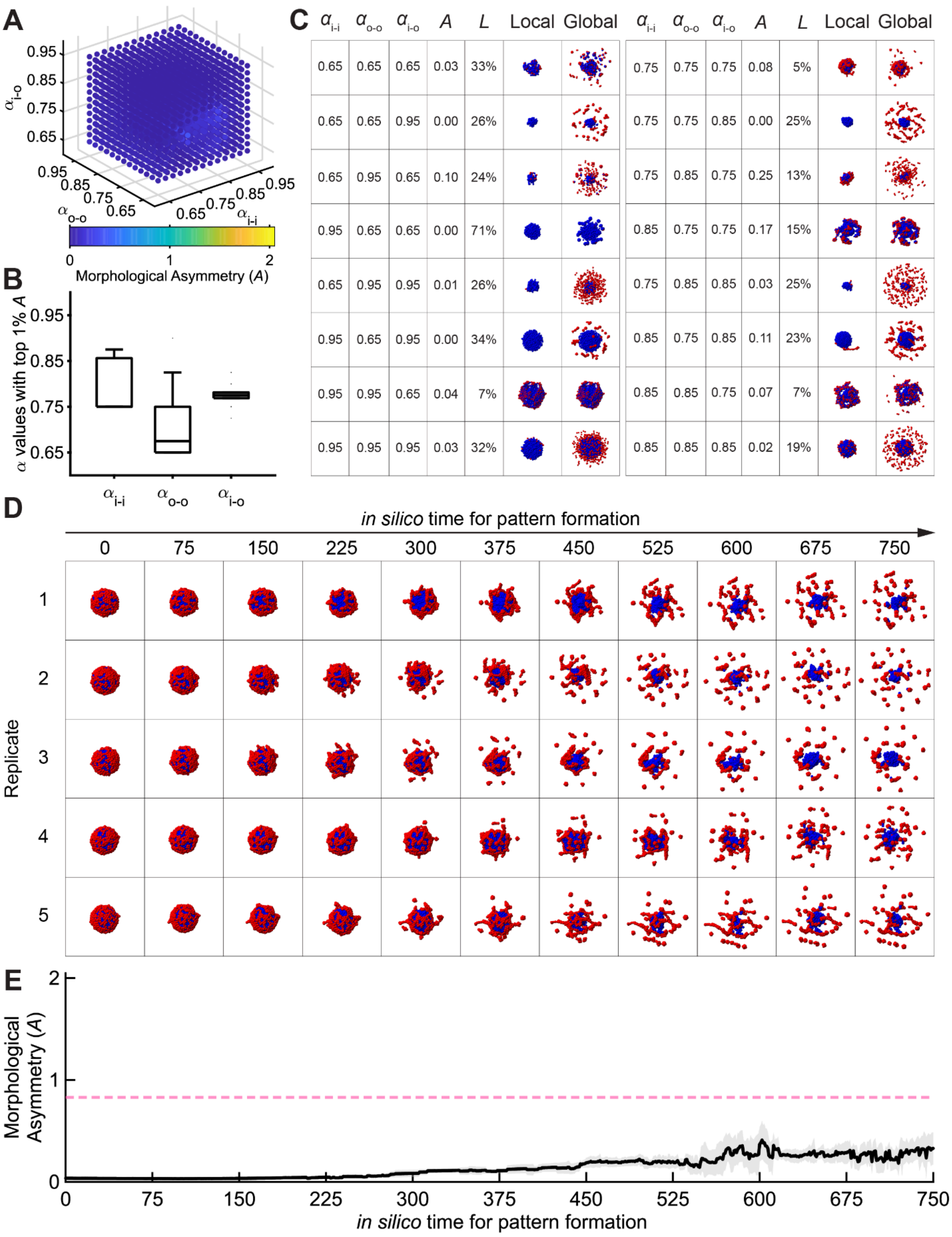
A
0.95
0.85
0.75
0.65
α_i-o
α_o-o
α_i-i
Morphological Asymmetry (A)
0
1
2
B
α values with top 1% A
α_i-i
α_o-o
α_i-o
C
α_i-i α_o-o α_i-o A L Local Global
0.65 0.65 0.65 0.03 33%
0.65 0.65 0.95 0.00 26%
0.65 0.95 0.65 0.10 24%
0.95 0.65 0.65 0.00 71%
0.65 0.95 0.95 0.01 26%
0.95 0.65 0.95 0.00 34%
0.95 0.95 0.65 0.04 7%
0.95 0.95 0.95 0.03 32%
0.75 0.75 0.75 0.08 5%
0.75 0.75 0.85 0.00 25%
0.75 0.85 0.75 0.25 13%
0.85 0.75 0.75 0.17 15%
0.75 0.85 0.85 0.03 25%
0.85 0.75 0.85 0.11 23%
0.85 0.85 0.75 0.07 7%
0.85 0.85 0.85 0.02 19%
D
in silico time for pattern formation
0 75 150 225 300 375 450 525 600 675 750
Replicate
1
2
3
4
5
E
Morphological Asymmetry (A)
in silico time for pattern formation

Figure S7. Morphogenetic landscape consisting of adhesion $\left(\alpha_{i-i}, \alpha_{o-o}, \alpha_{i-o}\right)$ and long-range attraction $\beta_{o\to o} = -0.15$.

(A) Heatmap showing morphological asymmetry (A) across the three short-range force parameters $\left(\alpha_{i-i}, \alpha_{o-o}, \alpha_{i-o}\right)$ (blue: low A; yellow: high A), revealing maximum $A = 0.328$.

(B) Boxplot showing the α value distribution for parameter combinations within the top 1% of A.

(C) Representative final morphologies evolved from a spherical aggregate, when the three short-range force parameters $\left(\alpha_{i-i}, \alpha_{o-o}, \alpha_{i-o}\right)$ are set as regular values. Results with extreme α values (0.65, mimicking strong adhesion; 0.95, mimicking weak adhesion) are shown on the left; results with moderate α values (0.75 and 0.85, mimicking moderate adhesion) are shown on the right. Here, the local pattern shows the largest aggregate with the most contacting cells; the global pattern shows the entirety of cells within the simulated system. Here, the local pattern shows the largest aggregate with the most contacting cells; the global pattern shows the entirety of cells within the simulated system.

(D) Morphological evolution from a spherical aggregate to weak or no symmetry breaking. Shown are five independent replicates with the highest morphological asymmetry observed under adhesion parameters $\left(\alpha_{i-i}, \alpha_{o-o}, \alpha_{i-o}\right) = (0.750, 0.650, 0.775)$.

(E) Morphological asymmetry curve over *in silico* time, plotted from the five independent replicates with the highest morphological asymmetry observed under short-range force parameters $\left(\alpha_{i-i}, \alpha_{o-o}, \alpha_{i-o}\right) = (0.750, 0.650, 0.775)$. Black solid line: mean; gray shade: standard deviation; pink dashed line: the maximum final A value when $\beta = 0$, corresponding to Figure 3.

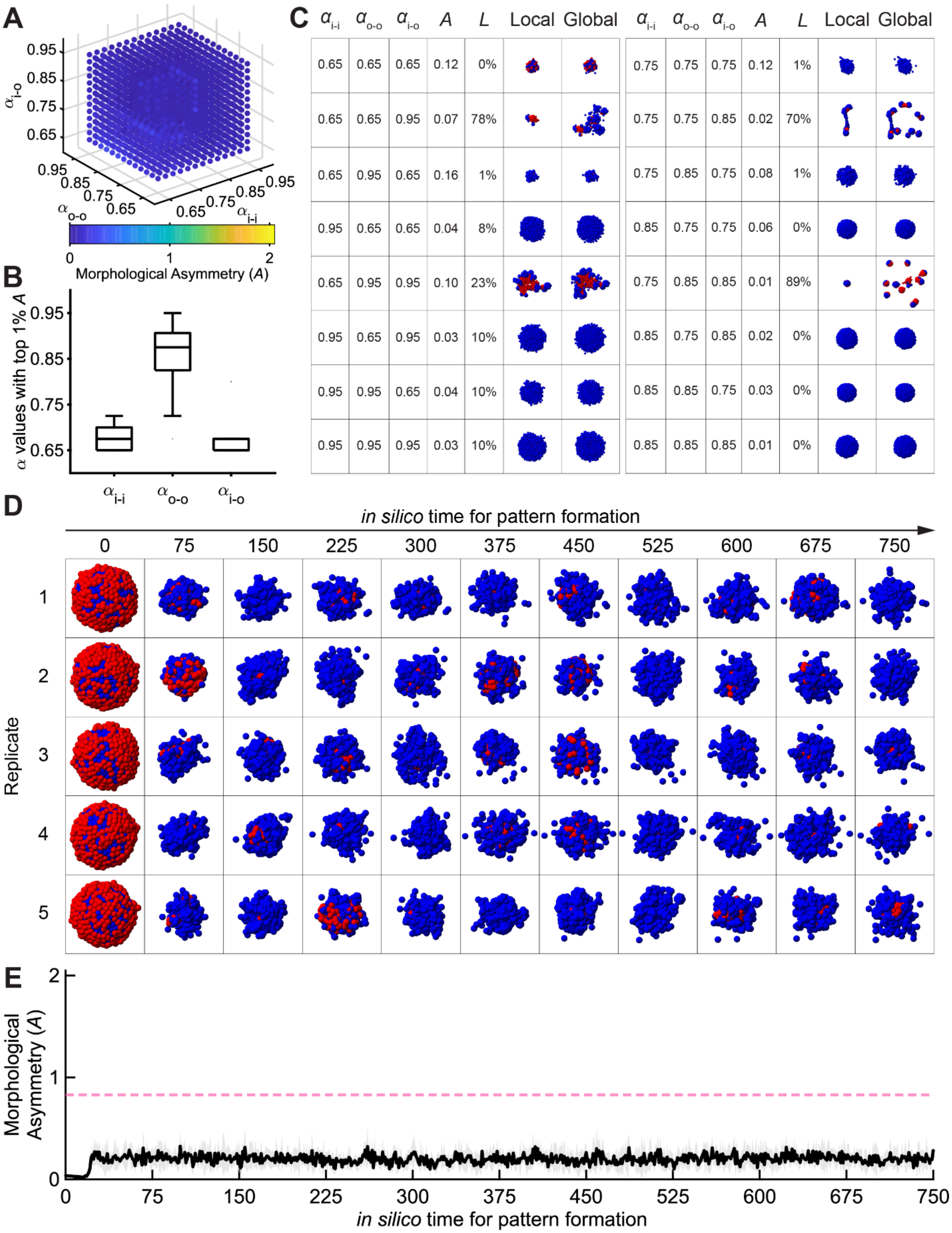
A
α_{i-o}
0.95
0.85
0.75
0.65
α_{o-o}
0.95
0.85
0.75
0.65
α_{i-i}
0.65
0.75
0.85
0.95
0
1
2
Morphological Asymmetry (A)
B
α values with top 1% A
0.95
0.85
0.75
0.65
α_{i-i}
α_{o-o}
α_{i-o}
C
α_{i-i} α_{o-o} α_{i-o} A L Local Global
0.65 0.65 0.65 0.12 0%
0.65 0.65 0.95 0.07 78%
0.65 0.95 0.65 0.16 1%
0.95 0.65 0.65 0.04 8%
0.65 0.95 0.95 0.10 23%
0.95 0.65 0.95 0.03 10%
0.95 0.95 0.65 0.04 10%
0.95 0.95 0.95 0.03 10%
α_{i-i} α_{o-o} α_{i-o} A L Local Global
0.75 0.75 0.75 0.12 1%
0.75 0.75 0.85 0.02 70%
0.75 0.85 0.75 0.08 1%
0.85 0.75 0.75 0.06 0%
0.75 0.85 0.85 0.01 89%
0.85 0.75 0.85 0.02 0%
0.85 0.85 0.75 0.03 0%
0.85 0.85 0.85 0.01 0%
D
in silico time for pattern formation
0
75
150
225
300
375
450
525
600
675
750
Replicate
1
2
3
4
5
E
Morphological Asymmetry (A)
2
1
0
0
75
150
225
300
375
450
525
600
675
750
in silico time for pattern formation

Figure S8. Morphogenetic landscape consisting of adhesion $\left(\alpha_{i-i}, \alpha_{o-o}, \alpha_{i-o}\right)$ and long-range attraction $\beta_{i\to o} = 0.15$

.

(A) Heatmap showing morphological asymmetry (A) across the three short-range force parameters $\left(\alpha_{i-i}, \alpha_{o-o}, \alpha_{i-o}\right)$ (blue: low A; yellow: high A), revealing maximum $A = 0.282$.

(B) Boxplot showing the α value distribution for parameter combinations within the top 1% of A.

(C) Representative final morphologies evolved from a spherical aggregate, when the three short-range force parameters $\left(\alpha_{i-i}, \alpha_{o-o}, \alpha_{i-o}\right)$ are set as regular values. Results with extreme α values (0.65, mimicking strong adhesion; 0.95, mimicking weak adhesion) are shown on the left; results with moderate α values (0.75 and 0.85, mimicking moderate adhesion) are shown on the right. Here, the local pattern shows the largest aggregate with the most contacting cells; the global pattern shows the entirety of cells within the simulated system.

(D) Morphological evolution from a spherical aggregate to weak or no symmetry breaking. Shown are five independent replicates with the highest morphological asymmetry observed under adhesion parameters $\left(\alpha_{i-i}, \alpha_{o-o}, \alpha_{i-o}\right) = (0.650, 0.875, 0.650)$.

(E) Morphological asymmetry curve over *in silico* time, plotted from the five independent replicates with the highest morphological asymmetry observed under short-range force parameters $\left(\alpha_{i-i}, \alpha_{o-o}, \alpha_{i-o}\right) = (0.650, 0.875, 0.650)$. Black solid line: mean; gray shade: standard deviation; pink dashed line: the maximum final A value when $\beta = 0$, corresponding to Figure 3.

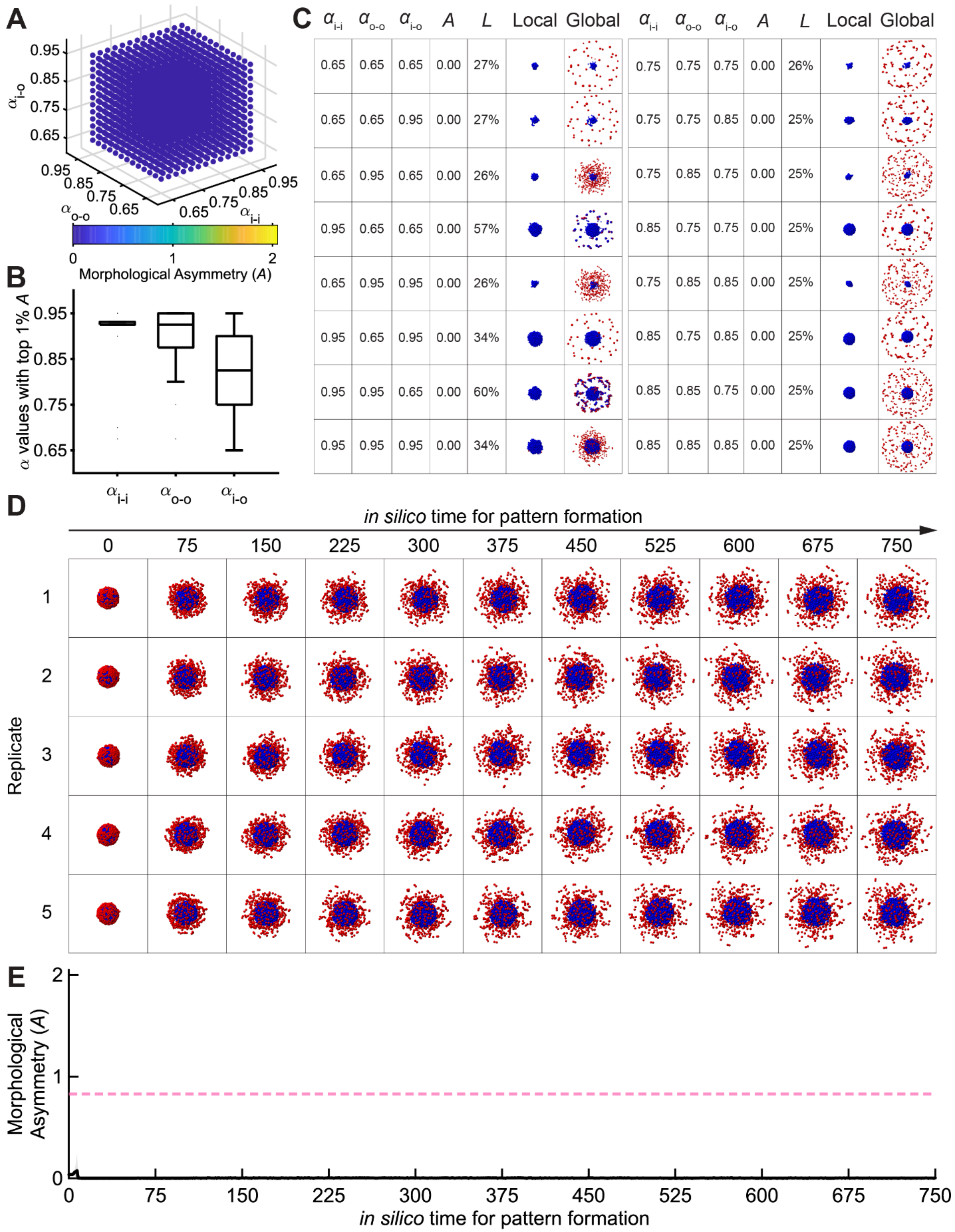
A
α_{i-o}
0.95
0.85
0.75
0.65
α_{o-o}
α_{i-i}
Morphological Asymmetry (A)
0 1 2
B
α values with top 1% A
α_{i-i} α_{o-o} α_{i-o}
C
α_{i-i} α_{o-o} α_{i-o} A L Local Global
0.65 0.65 0.65 0.00 27%
0.65 0.65 0.95 0.00 27%
0.65 0.95 0.65 0.00 26%
0.95 0.65 0.65 0.00 57%
0.65 0.95 0.95 0.00 26%
0.95 0.65 0.95 0.00 34%
0.95 0.95 0.65 0.00 60%
0.95 0.95 0.95 0.00 34%
0.75 0.75 0.75 0.00 26%
0.75 0.75 0.85 0.00 25%
0.75 0.85 0.75 0.00 25%
0.85 0.75 0.75 0.00 25%
0.75 0.85 0.85 0.00 25%
0.85 0.75 0.85 0.00 25%
0.85 0.85 0.75 0.00 25%
0.85 0.85 0.85 0.00 25%
D
in silico time for pattern formation
0 75 150 225 300 375 450 525 600 675 750
Replicate
1 2 3 4 5
E
Morphological Asymmetry (A)
2 1 0
0 75 150 225 300 375 450 525 600 675 750
in silico time for pattern formation

Figure S9. Morphogenetic landscape consisting of adhesion $\left(\alpha_{i-i}, \alpha_{o-o}, \alpha_{i-o}\right)$ and long-range attraction $\beta_{i\rightarrow o} =- 0.15$.

(A) Heatmap showing morphological asymmetry (A) across the three short-range force parameters $\left(\alpha_{i-i}, \alpha_{o-o}, \alpha_{i-o}\right)$ (blue: low A; yellow: high A), revealing maximum $A = 0.003$.

(B) Boxplot showing the α value distribution for parameter combinations within the top 1% of A.

(C) Representative final morphologies evolved from a spherical aggregate, when the three short-range force parameters $\left(\alpha_{i-i}, \alpha_{o-o}, \alpha_{i-o}\right)$ are set as regular values. Results with extreme α values (0.65, mimicking strong adhesion; 0.95, mimicking weak adhesion) are shown on the left; results with moderate α values (0.75 and 0.85, mimicking moderate adhesion) are shown on the right. Here, the local pattern shows the largest aggregate with the most contacting cells; the global pattern shows the entirety of cells within the simulated system.

(D) Morphological evolution from a spherical aggregate to weak or no symmetry breaking. Shown are five independent replicates with the highest morphological asymmetry observed under adhesion parameters $\left(\alpha_{i-i}, \alpha_{o-o}, \alpha_{i-o}\right) = (0.950, 0.950, 0.900)$.

(E) Morphological asymmetry curve over *in silico* time, plotted from the five independent replicates with the highest morphological asymmetry observed under short-range force parameters $\left(\alpha_{i-i}, \alpha_{o-o}, \alpha_{i-o}\right) = (0.950, 0.950, 0.900)$. Black solid line: mean; gray shade: standard deviation; pink dashed line: the maximum final A value when $\beta = 0$, corresponding to Figure 3.

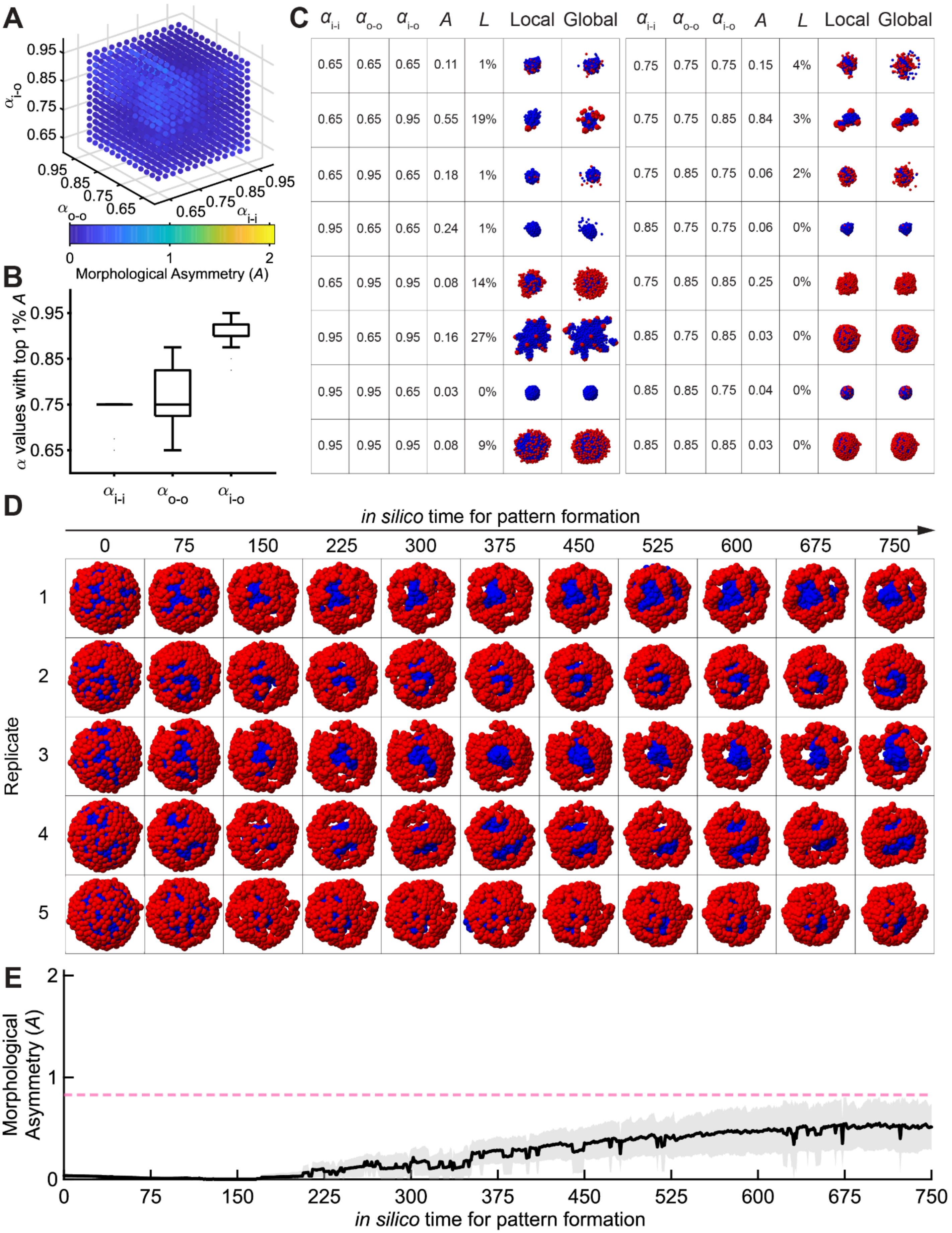
A
α_{i-o}
0.95
0.85
0.75
0.65
α_{o-o}
α_{i-i}
0 1 2
Morphological Asymmetry (A)
B
α values with top 1% A
α_{i-i} α_{o-o} α_{i-o}
C
α_{i-i} α_{o-o} α_{i-o} A L Local Global
0.65 0.65 0.65 0.11 1%
0.65 0.65 0.95 0.55 19%
0.65 0.95 0.65 0.18 1%
0.95 0.65 0.65 0.24 1%
0.65 0.95 0.95 0.08 14%
0.95 0.65 0.95 0.16 27%
0.95 0.95 0.65 0.03 0%
0.95 0.95 0.95 0.08 9%
0.75 0.75 0.75 0.15 4%
0.75 0.75 0.85 0.84 3%
0.75 0.85 0.75 0.06 2%
0.85 0.75 0.75 0.06 0%
0.75 0.85 0.85 0.25 0%
0.85 0.75 0.85 0.03 0%
0.85 0.85 0.75 0.04 0%
0.85 0.85 0.85 0.03 0%
D
in silico time for pattern formation
0 75 150 225 300 375 450 525 600 675 750
Replicate
1
2
3
4
5
E
Morphological Asymmetry (A)
0 1 2
0 75 150 225 300 375 450 525 600 675 750
in silico time for pattern formation

Figure S10. Morphogenetic landscape consisting of adhesion $\left(\alpha_{i-i}, \alpha_{o-o}, \alpha_{i-o}\right)$ and long-range attraction $\beta_{o\rightarrow i} = 0.15$.

(A) Heatmap showing morphological asymmetry (A) across the three short-range force parameters $\left(\alpha_{i-i}, \alpha_{o-o}, \alpha_{i-o}\right)$ (blue: low A; yellow: high A), revealing maximum $A = 0.513$.

(B) Boxplot showing the α value distribution for parameter combinations within the top 1% of A.

(C) Representative final morphologies evolved from a spherical aggregate, when the three short-range force parameters $\left(\alpha_{i-i}, \alpha_{o-o}, \alpha_{i-o}\right)$ are set as regular values. Results with extreme α values (0.65, mimicking strong adhesion; 0.95, mimicking weak adhesion) are shown on the left; results with moderate α values (0.75 and 0.85, mimicking moderate adhesion) are shown on the right. Here, the local pattern shows the largest aggregate with the most contacting cells; the global pattern shows the entirety of cells within the simulated system.

(D) Morphological evolution from a spherical aggregate to weak or no symmetry breaking. Shown are five independent replicates with the highest morphological asymmetry observed under adhesion parameters $\left(\alpha_{i-i}, \alpha_{o-o}, \alpha_{i-o}\right) = (0.750, 0.750, 0.950)$.

(E) Morphological asymmetry curve over *in silico* time, plotted from the five independent replicates with the highest morphological asymmetry observed under short-range force parameters $\left(\alpha_{i-i}, \alpha_{o-o}, \alpha_{i-o}\right) = (0.750, 0.750, 0.950)$. Black solid line: mean; gray shade: standard deviation; pink dashed line: the maximum final A value when $\beta = 0$, corresponding to Figure 3.

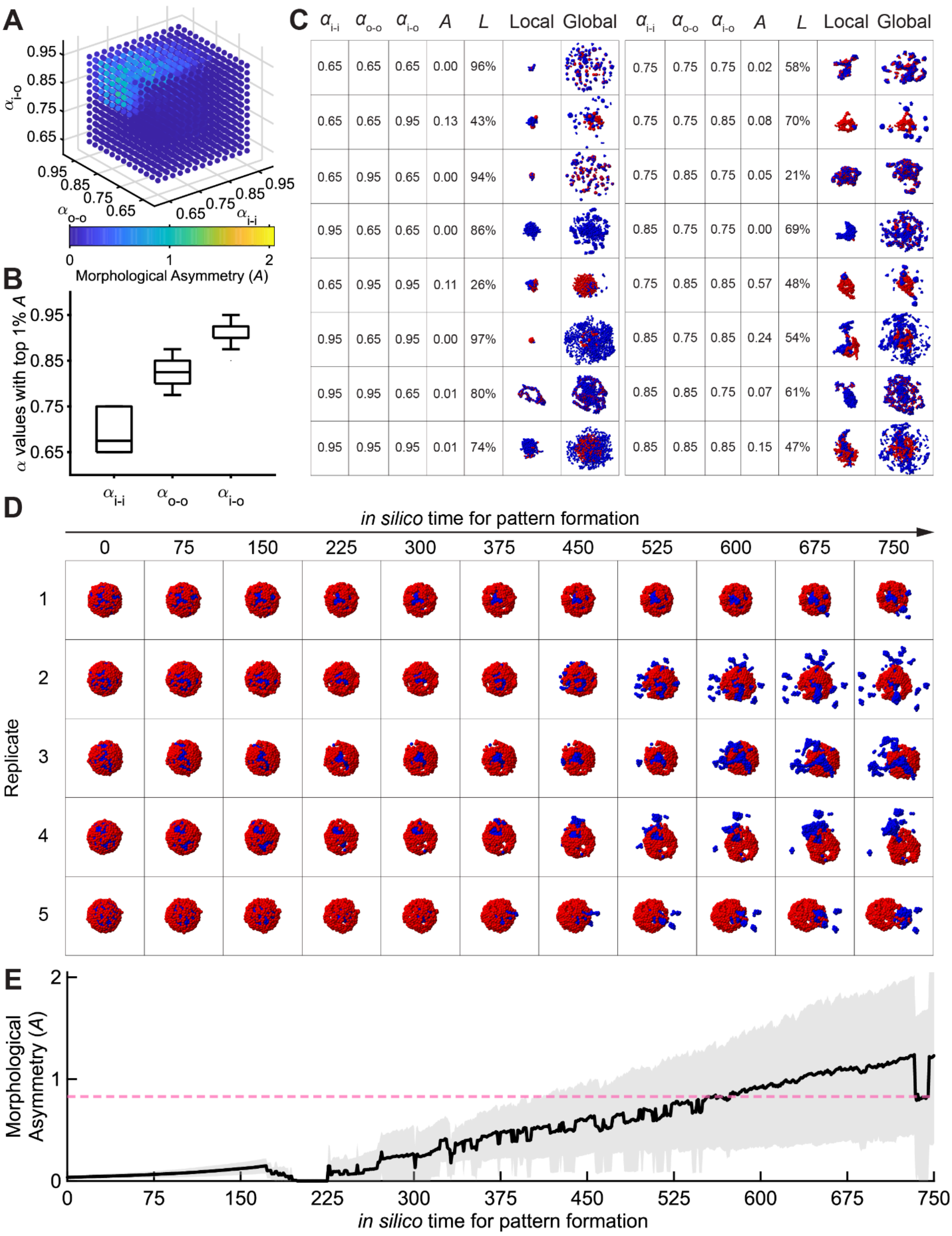

A
0.95
0.85
0.75
0.65
$\alpha_{i\text{-}o}$
$\alpha_{o\text{-}o}$
$\alpha_{i\text{-}i}$
0
1
2
Morphological Asymmetry (A)
B
α values with top 1% A
$\alpha_{i\text{-}i}$
$\alpha_{o\text{-}o}$
$\alpha_{i\text{-}o}$
C
$\alpha_{i\text{-}i}$ $\alpha_{o\text{-}o}$ $\alpha_{i\text{-}o}$ A L Local Global
0.65 0.65 0.65 0.00 96%
0.65 0.65 0.95 0.13 43%
0.65 0.95 0.65 0.00 94%
0.95 0.65 0.65 0.00 86%
0.65 0.95 0.95 0.11 26%
0.95 0.65 0.95 0.00 97%
0.95 0.95 0.65 0.01 80%
0.95 0.95 0.95 0.01 74%
0.75 0.75 0.75 0.02 58%
0.75 0.75 0.85 0.08 70%
0.75 0.85 0.75 0.05 21%
0.85 0.75 0.75 0.00 69%
0.75 0.85 0.85 0.57 48%
0.85 0.75 0.85 0.24 54%
0.85 0.85 0.75 0.07 61%
0.85 0.85 0.85 0.15 47%
D
in silico time for pattern formation
0 75 150 225 300 375 450 525 600 675 750
Replicate
1
2
3
4
5
E
Morphological Asymmetry (A)
0
1
2
0 75 150 225 300 375 450 525 600 675 750
in silico time for pattern formation

Figure S11. Morphogenetic landscape consisting of adhesion $\left(\alpha_{i-i}, \alpha_{o-o}, \alpha_{i-o}\right)$ and long-range attraction $\beta_{o\rightarrow i} =- 0.15$.

(A) Heatmap showing morphological asymmetry (A) across the three short-range force parameters $\left(\alpha_{i-i}, \alpha_{o-o}, \alpha_{i-o}\right)$ (blue: low A; yellow: high A), revealing maximum $A = 1.229$.

(B) Boxplot showing the α value distribution for parameter combinations within the top 1% of A.

(C) Representative final morphologies evolved from a spherical aggregate, when the three short-range force parameters $\left(\alpha_{i-i}, \alpha_{o-o}, \alpha_{i-o}\right)$ are set as regular values. Results with extreme α values (0.65, mimicking strong adhesion; 0.95, mimicking weak adhesion) are shown on the left; results with moderate α values (0.75 and 0.85, mimicking moderate adhesion) are shown on the right. Here, the local pattern shows the largest aggregate with the most contacting cells; the global pattern shows the entirety of cells within the simulated system.

(D) Morphological evolution from a spherical aggregate to weak or no symmetry breaking. Shown are five independent replicates with the highest morphological asymmetry observed under adhesion parameters $\left(\alpha_{i-i}, \alpha_{o-o}, \alpha_{i-o}\right) = (0.750, 0.800, 0.925)$.

(E) Morphological asymmetry curve over *in silico* time, plotted from the five independent replicates with the highest morphological asymmetry observed under short-range force parameters $\left(\alpha_{i-i}, \alpha_{o-o}, \alpha_{i-o}\right) = (0.750, 0.800, 0.925)$. Black solid line: mean; gray shade: standard deviation; pink dashed line: the maximum final A value when $\beta = 0$, corresponding to Figure 3.

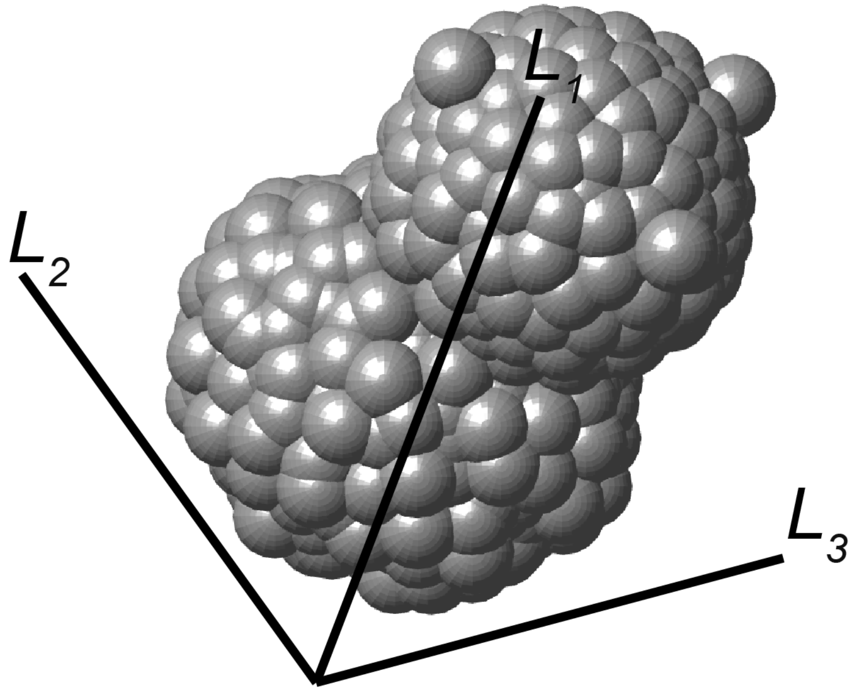

Figure S12. Schematic diagram for determining the three principal axes of a *DevSim*-produced 3D object. (L_1, L_2, L_3) denotes the lengths of three orthogonal axes (corresponding to the longest, intermediate, and shortest dimensions respectively) of the oriented bounding box (OBB) fitted to the 3D object [Guan et al. *Membranes* 2024].

Supplemental Table

Table S1. Detailed information of simulated morphogenetic landscape considering all basic short-range force parameters $\left(\alpha_{i-i}, \alpha_{o-o}, \alpha_{i-o}\right)$ and additional long-range force parameters β.

Table S2. Detailed information of shape description implemented in *DevSim* platform. $\left(L_1, L_2, L_3\right)$ denotes the lengths of three orthogonal axes (corresponding to the longest, intermediate, and shortest dimensions respectively) of the oriented bounding box (OBB) fitted to the 3D object [Guan et al. *Membranes* 2024].

Supplemental Movie

Movie S1. Bright-field time-lapse recording of four human gastruloids from the same batch, with or without CHIR treatment on Day 0.

Movie S2. Time-lapse simulations of five replicates under differential adhesion $\left(\alpha_{i-i}, \alpha_{o-o}, \alpha_{i-o}\right) = (0.775, 0.950, 0.875)$ and long-range attraction $\beta_{o\to o} = 0.135$. Here, initially outer and inner cells are shown in red and blue respectively.

Movie S3. Time-lapse simulations of five replicates under only differential adhesion $\left(\alpha_{i-i}, \alpha_{o-o}, \alpha_{i-o}\right) = (0.775, 0.950, 0.875)$. Here, initially outer and inner cells are shown in red and blue respectively.

Movie S4. Time-lapse simulation of genetic-mechanical regulatory network giving rise to a morphogenetic procedure in which outer cells migrate along the periphery and converge.

Movie S5. Step-by-step instruction for *DevSim* platform.

Movie S6. *DevSim*-exported video for a morphogenetic procedure in which a layered pattern is formed.

Movie S7. *DevSim*-exported video for a morphogenetic procedure in which a bilobed pattern is formed.

Movie S8. *DevSim*-exported video for a morphogenetic procedure in which a multilobed pattern is formed.

Supplemental Text 1

DevSim User Guidebook

Purpose

DevSim is a MATLAB app for simulating multicellular development with coarse-grained cells moving in an overdamped medium, gene circuits governed by Hill functions, and long- and short-range cell-to-cell interactions. It's designed to help users explore a range of developmental patterns (e.g., symmetry breaking, aggregation, peripheral patterning), visualize outcomes, and export measurements (gene expression over time, 3D shape descriptions).

Availability and Citation

Software name: DevSim

Distribution: https://github.com/hormoz-lab/DevSim.git

How to cite (placeholder): "DevSim (Developmental-Simulator), version 1.0. MATLAB app and example templates. https://github.com/hormoz-lab/DevSim.git"

Contact: tgshields@college.harvard.edu

License: Public use.

System Requirements

MATLAB: created/tested on **R2024b**; should run on recent releases.

Toolboxes: Parallel Computing Toolbox (for parfor).

OS: Windows & macOS tested (Linux not yet tested).

Excel editing: The "Edit …" buttons attempt to open Microsoft Excel. If Excel isn't present, open the .xlsx files manually in the user's editor of choice.

Hardware: No GPU required. With the default settings (~1,350 cells; 8 sims; dt=0.2; Tmax=50), an M-series MacBook Pro (M4) completes in ~2 minutes. Any modern Intel i5 or better, or any Apple-silicon (M-series) Mac is fine

Folder layout and files inside DevSim

```
DevSim/
    DevSim_GUI.m
    RunSimulation.m
    Active/
        UserParams.xlsx
        GeneRegulatoryNetwork.xlsx
        GeneParameters.xlsx
    Templates/
        SymmetryBreakingTemplate/
            UserParams.xlsx
            GeneRegulatoryNetwork.xlsx
```

GeneParameters.xlsx

OUTPUT1/

OUTPUT2/

…

Backups/

Template Pack

A **template pack** is simply a folder containing the three .xlsx files above. Use the "Export Template" button in the *DevSim* GUI to create one; use the "Load Template" button to copy one into Active/. A Default Symmetry-Breaking Template Pack will be provided.

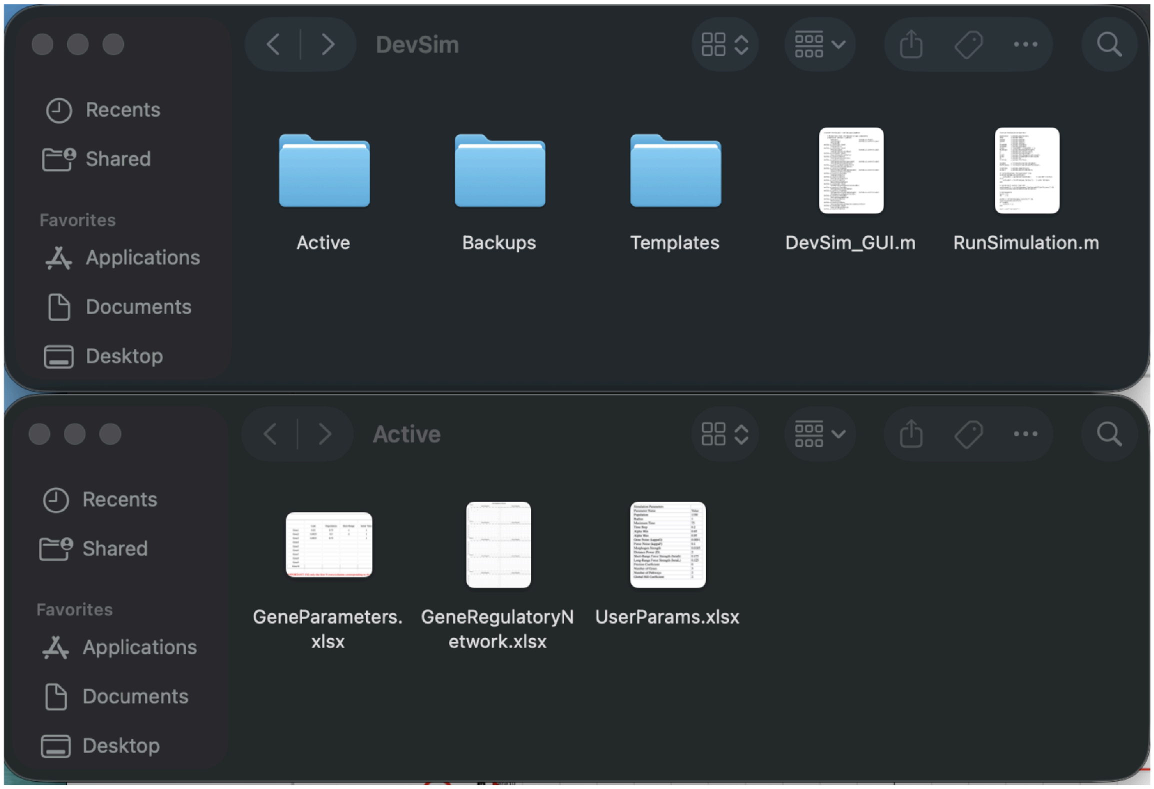

Figure G1. DevSim/ directory (top) and Active/ directory (bottom).

Step-by-step Tutorial

(A) Launch *DevSim*

1. Place the DevSim folder somewhere convenient. Ensure all components exist: DevSim/Active/; DevSim/DevSim_GUI.m; DevSim/RunSimulation.m; DevSim/Templates/.

2. Open MATLAB version: R2024b (older versions may not be able to support *DevSim*). In MATLAB, set the Current Folder to the DevSim root.

3. Run DevSim_GUI to open the app.

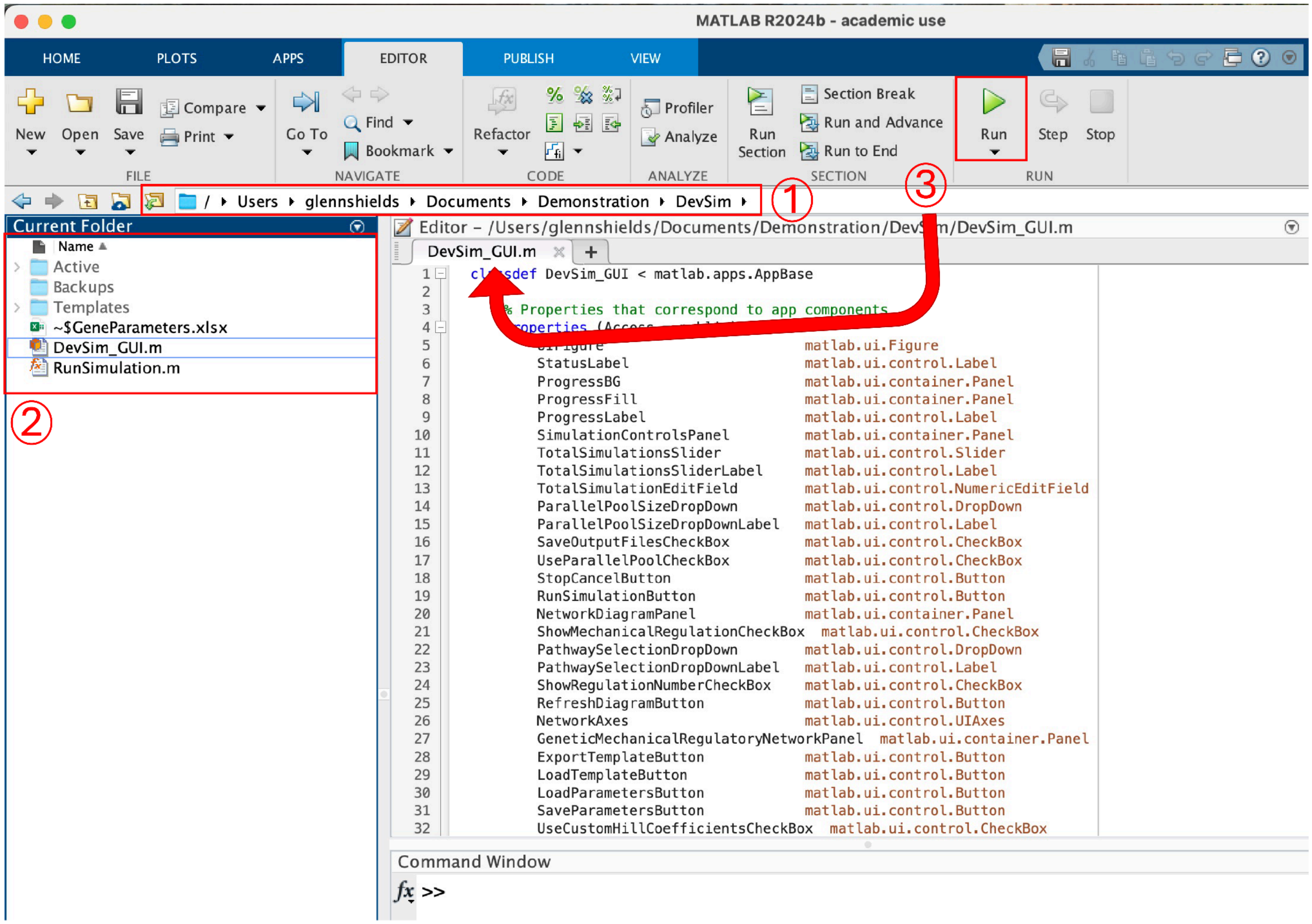

Figure G2. Launching *DevSim* (steps for section A). ① Open MATLAB R2024b (older versions may not support *DevSim*). In MATLAB, set the Current Folder to the *DevSim* root. ② Ensure all folders are properly nested, double-click on "DevSim_GUI.m" to load the script into the MATLAB window. ③ Make sure the open script is "DevSim_GUI.m" and press Run to open the *DevSim* GUI.

(B) Start from known default parameters

1. When opened, *DevSim* loads default parameters. At any time, the user can press "Reset Parameters" to reset to the default parameters in the *DevSim* Graphical User Interface (GUI); pressing "Reset Parameters" will not affect the Active/ folder or the Active Excel workbooks. The default parameters in the GUI are as follows:

Population Size = 1350; Radius = 1; Maximum Time = 50; Time Step (dt) = 0.2; Alpha Min = 0.65; Alpha Max = 0.95; Gene Noise (kappaG) = 0.0001; Force Noise (kappaF) = 0.1; Morphogen Strength = 0.0185; Distance Power (D) = 2; Short-Range Force Strength (betaS) = 0.175; Long-Range Force Strength (betaL) = 0.125; Friction Coefficient = 0; Number of Genes = 3; Number of Pathways = 2; Global Hill Coefficient = 2.

Note: Parameters have minimal bounds to maximize flexibility; it is recommended to start from the defaults before exploring the large parameter space.

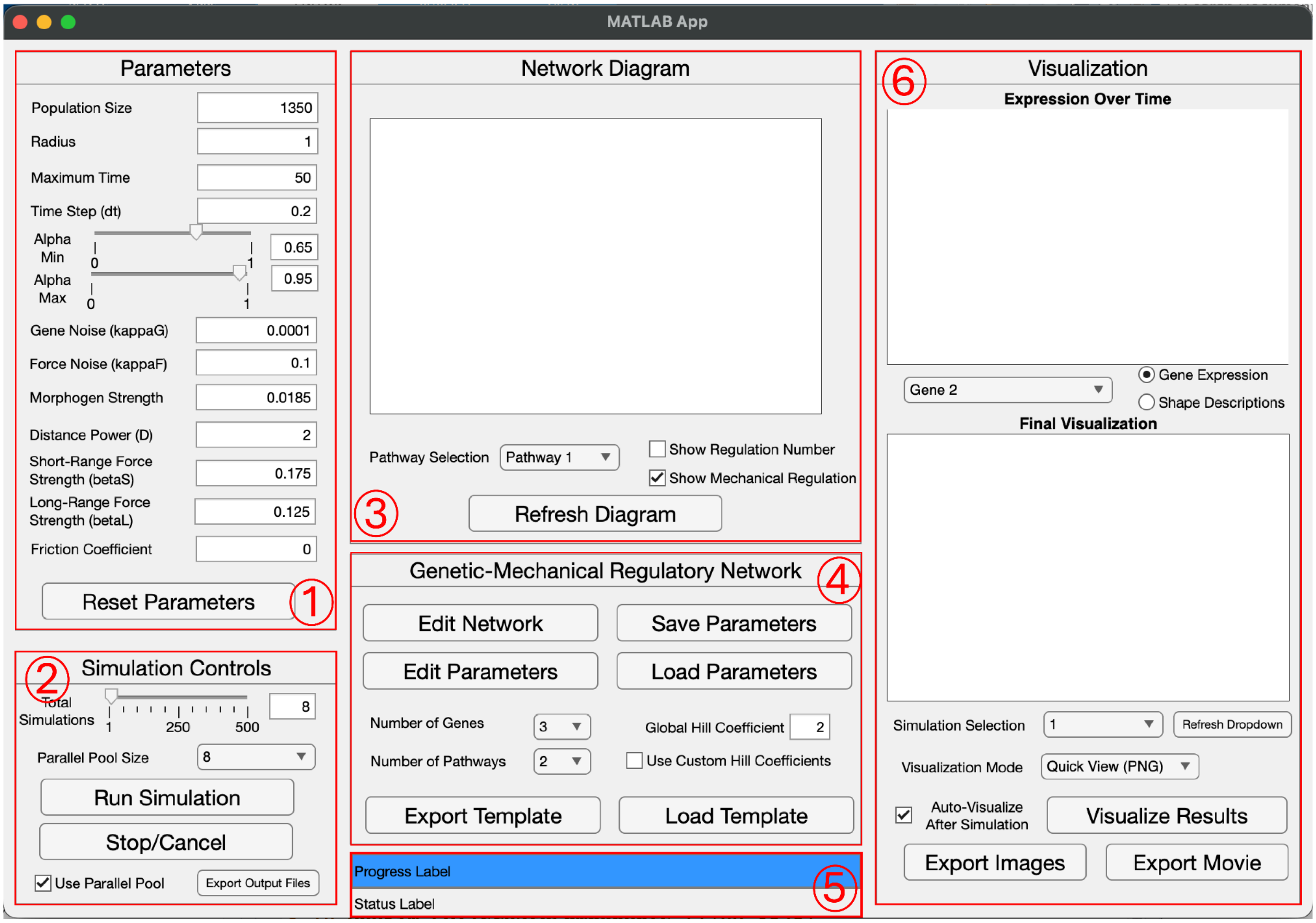

Figure G3. *DevSim* GUI with panels and important features highlighted in red and numbered. ① Parameters panel. ② Simulation Controls panel. ③ Network Diagram panel ④ Genetic-Mechanical Regulatory Network Pane (GMRN). ⑤ Progress Bar, Progress Label, and Status Label. ⑥ Visualization panel.

(C) Decide how many simulations and whether to use parallel

In the Simulation Controls panel:

1. Set Total Simulations (1-500)
2. Toggle Use Parallel Pool and choose Pool Size (1-8) if the user wants to use simultaneous runs

Warning (MATLAB Online users): MATLAB Online doesn't support parallel pools - uncheck Use Parallel Pool box. Otherwise *DevSim* is fully compatible with the online version of MATLAB.

Canceling: "Stop/Cancel" button halts the simulation after the current batch; it does not interrupt an active parfor iteration. To stop immediately during a parfor, the user may need to close the app/MATLAB directly.

(D) Inspect or set the size of the Genetic-Mechanical Regulatory Network (GMRN)

1. Choose Number of Genes (1-10) and Number of Pathways (1-10) in Genetic-Mechanical Regulatory Network panel.
2. Set Global Hill Coefficient (used everywhere unless overridden per-edge)
3. Check "Use Custom Hill Coefficients" to set per-edge Hill coefficients in the Excel workbook.

Design Choice: *DevSim* supports up to 10 genes and 10 regulatory pathways. Pathways are visualized in distinct colors (Pathway 1 = blue, 2 = red, 3 = green, …).

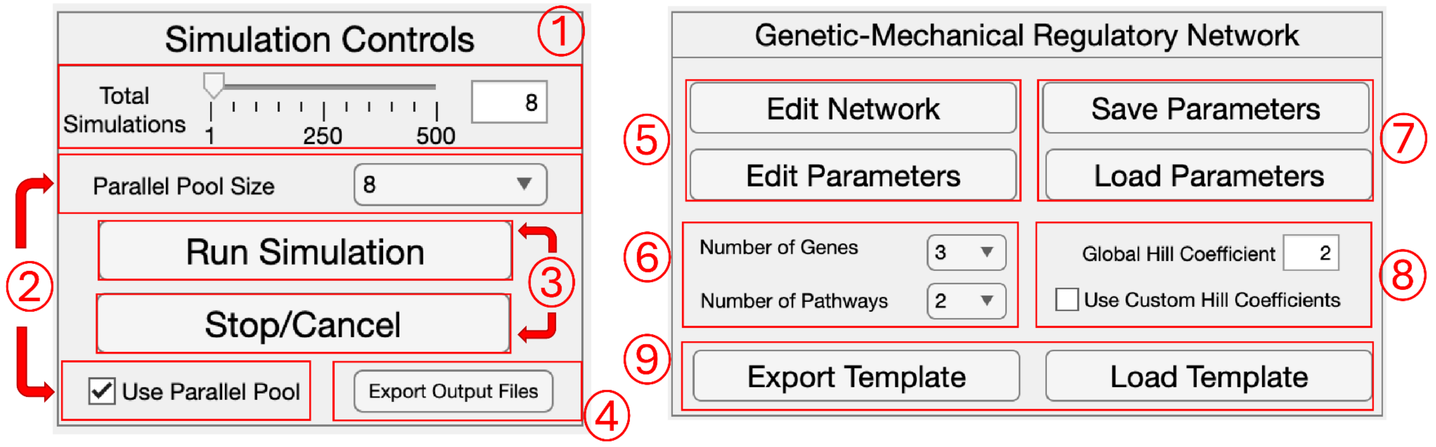

Figure G4. *DevSim* Simulation Controls (Left) and Genetic-Mechanical Regulatory Network (Right) panels. ① Total Simulations Slider and Edit Field. Users can enter the number of total simulations they want to run from 1 - 500. ② Parallel Pool Size Dropdown (top) and Use Parallel Pool checkbox (bottom). To use Parallel Pool, the Use Parallel Pool checkbox should be checked, and the user will pick the number of simultaneous simulations to run via MATLAB parfor in the parallel pool size dropdown from 1 - 8. If total simulations exceeds parallel pool size, the simulations will run in batches the size of the parallel pool size value. ③ Run Simulation button (top) pressed when user is satisfied with all the parameters and Genetic-Mechanical Regulatory Network and wants to run the simulation. Stop/Cancel button (bottom) pressed when user wants to stop the simulation after the current batch (will not stop simulation during a parfor loop). ④ Export Output Files button. Users can press this button to export the entire experiment into a single parent folder for archiving or sharing, they will first choose a destination directory; then when prompted, name their Parent folder. ⑤ Edit Network button (top) will open GeneRegulatoryNetwork.xlsx file via the Active folder directory when pressed. Edit Parameters button (bottom) will open GeneParameters.xlsx file via the Active folder directory when pressed. ⑥ Number of Genes Dropdown (top) users can choose the number of genes 1 - 10 they would like to simulate. Number of Pathways (bottom) users can choose the number of parallel pathways 1 - 10 they would like to simulate. ⑦ Save Parameters Button (top) will store the current user parameter values entered in the Parameters panel inside of the *DevSim* GUI and also the Number of Genes, Number of Pathways, and Global Hill coefficient value entered in the GMRN panel into the UserParams.xlsx file via the Active folder Directory when pressed. Load Parameters Button (bottom) will load the user parameter values inside the UserParams.xlsx file in the Active folder Directory into the Parameters and GMRN panels. ⑧ Global Hill Coefficient Edit Field (top) and Use Custom Hill Coefficients (bottom). When "Use Custom Hill Coefficients" is checked, *DevSim* will read the user entries inside of the Active/GeneRegulatoryNetwork.xlsx in the HillCoeffs Sheet. ⑨ Export Template button (left) when pressed will prompt users to create a name for the template folder and once entered will transfer the Active file directory's GeneParameters.xlsx, GeneRegulatoryNetwork.xlsx, and UserParams.xlsx into the user named template folder under the Templates/ file directory. Load Template button (top) when pressed will prompt users to select a Template via the Templates/ file directory; once selected, that template will be loaded into *DevSim* via replacing the Active file directory's files with the Template's files.

(E) Edit the network (Excel): internal versus external regulation

1. Click Edit Network button in the GMRN panel to open **Active/GeneRegulatoryNetwork.xlsx.**

2. For each pathway p, there are two gene number by gene number (NxN) blocks:

- Internal Regulation (left block; solid edges in the GUI diagram).
- External (morphogen-mediated) regulation (right block; dashed edges in the GUI diagram)

3. Directionality: Rows = targets (receivers), Columns = sources (senders).

4. Enter weights: 0 = no edge (can also leave cell empty), > 0 = activation, < 0 = inhibition

5. If Use Custom Hill Coefficients is checked, open the HillCoeffs sheet and enter values > 0 only where the user want to override the global H (leave blank to inherit the global value).

6. Save the workbook, go back to *DevSim*, and click the "Refresh Diagram" button in the Network Diagram panel to visualize the edits

IMPORTANT: If the workbook is not saved before going back to *DevSim*, none of the edits made in the workbook will be applied to *DevSim* or the current simulation.

Edge semantics (meaning of entries):

- Weight magnitude is the regulatory strength. The sign sets activation (positive) vs inhibition (negative).
- The Hill coefficient H is the sigmoid steepness: Large H = switch-like logic; small H = more gradual logic

Each nonzero edge weight is interpreted as the signed input strength to a sigmoidal regulation term (Hill function); the Hill coefficient controls steepness, not the sign.

IMPORTANT: The diagram only displays up to the "Number of Genes" and "Number of Pathways" set in the GUI. Entries outside those ranges exist in the file, but won't be plotted or simulated unless the user increases the "Number of Genes" and/or the "Number of Pathways" inside of the GUI.

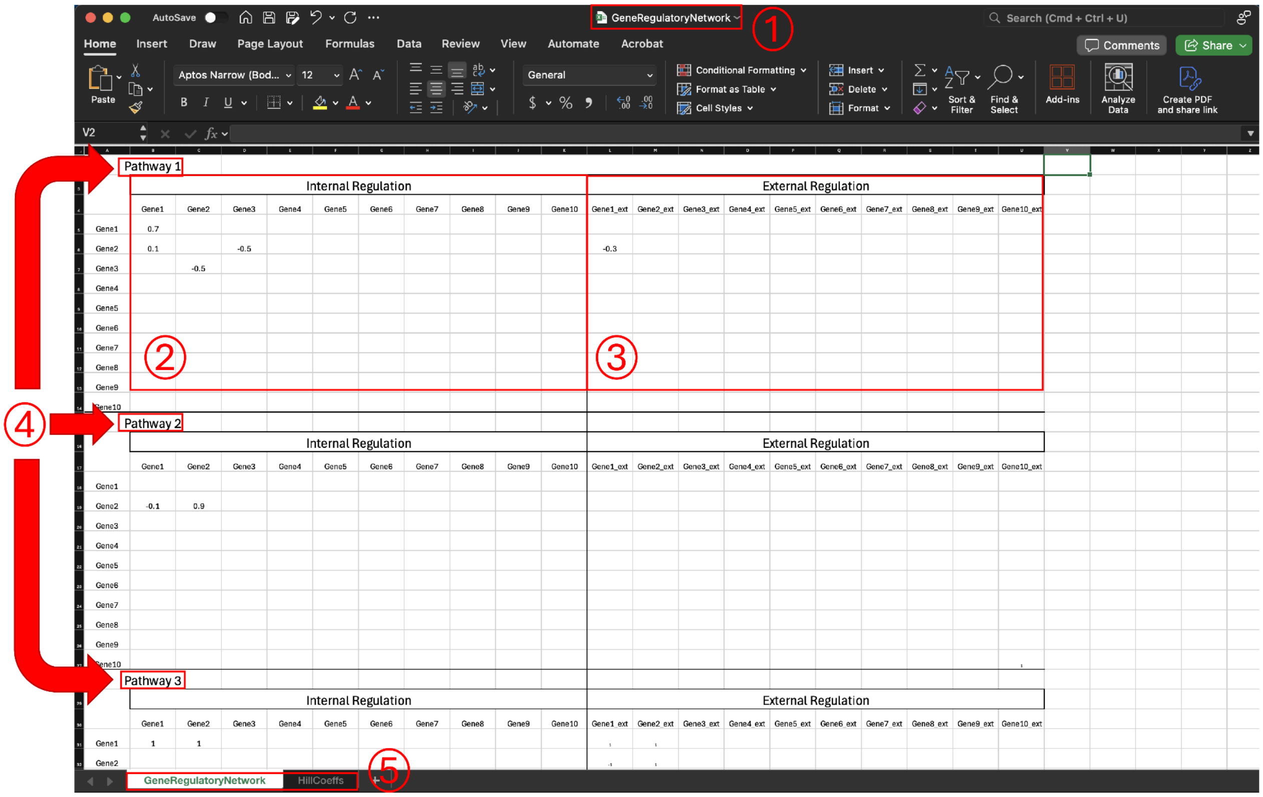

Figure G5. GeneRegulatoryNetwork.xlsx shown and can be edited by the user to change the *DevSim* GMRN. ① GeneRegulatoryNetwork.xlsx should open when pressing the Edit Network button found inside the *DevSim* GUI in the GMRN panel. ② Internal Regulation encoded NxN block for pathway 1. Entries in this block will show up as solid lines inside of the *DevSim* GUI for pathway 1 (blue) where the rows are the targets and the columns are the sources. ③ External (Morphogen) Regulation encoded NxN block for pathway 1. Entries in this block will show up as dashed lines inside of the *DevSim* GUI for pathway 1 (blue) where the rows are the targets and the columns are the sources. ④ Internal and External Regulation block logic are repeated for All 10 pathways, 3 of which are shown in the current window ⑤ In order to add custom Hill Coefficients for GMRN connections, the GeneRegulatoryNetwork sheet's format is duplicated on a separate sheet named HillCoeffs, where all the custom Hill Coefficients will be stored for their corresponding connections.

Optional Network Diagram viewing modes

Toggle "Show Mechanical Regulation" in the Network Diagram panel to display gray connections to:

- A (adhesion/short-range), C_A (chemotaxis attraction), C_R (chemotaxis repulsion)
- These summarize how gene-dependent mechanical channels feed into forces – the unique logic of *DevSim*

Toggle "Show Regulation Number" in the Network Diagram panel to display, for each node (gene), incoming and outgoing counts for internal and external edges (self-loops included).

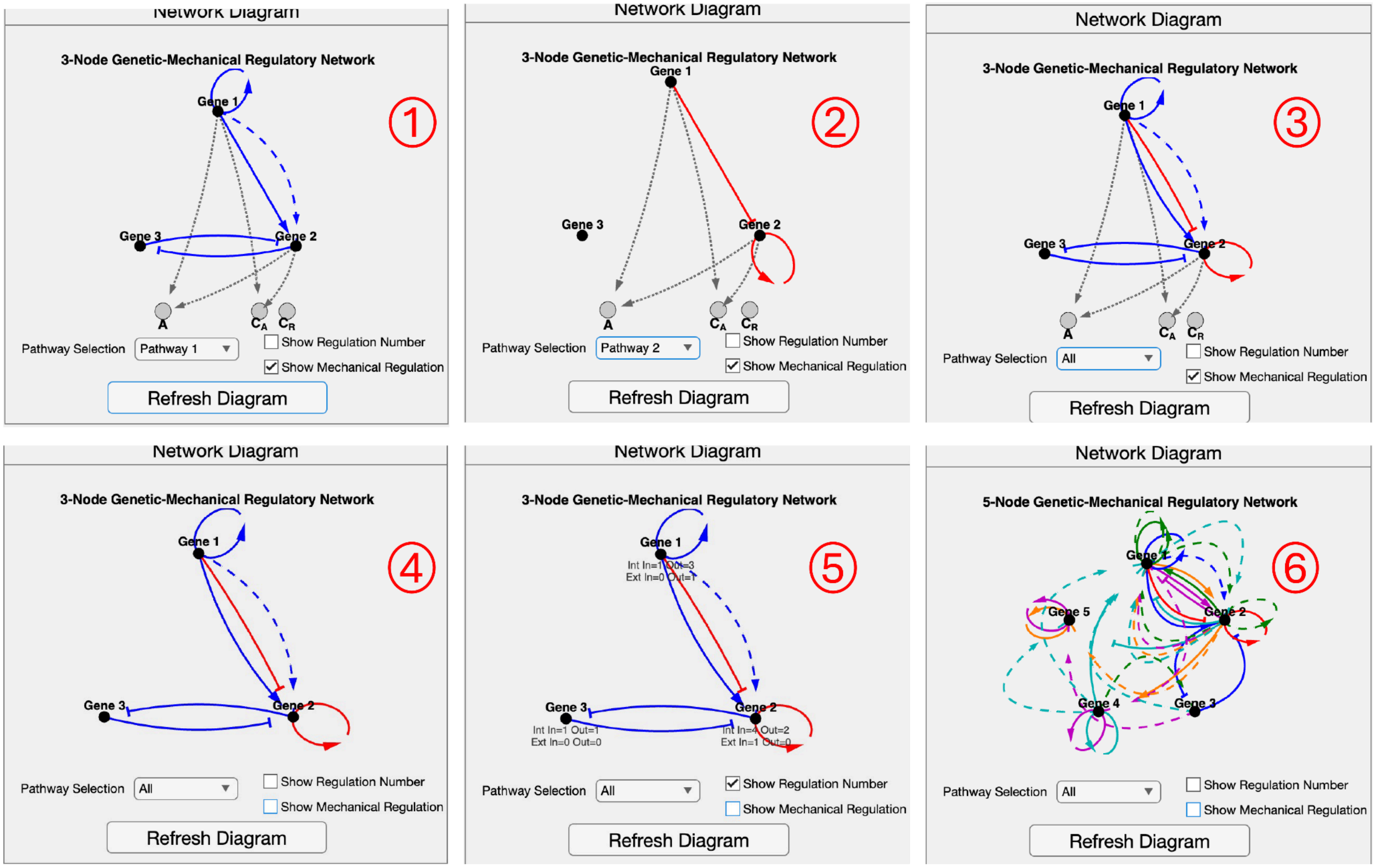

Figure G6. Six examples of *DevSim* GMRN diagram are shown, visualized in the Network Diagram panel in order showcase the graphing features and optional viewing modes. Single pathways or multiple pathways can be visualized: ① Default GMRN pathway 1 connections are shown by selecting Pathway 1 in the Pathway Selection Dropdown which are

represented by blue connections. ② Default GMRN pathway 2 connections are shown by selecting Pathway 2 in the Pathway Selection Dropdown which are represented by red connections. ③ Default GMRN shown with both pathway 1 and pathway 2 by selecting “All” in the Pathway Selection Dropdown. ④ Gray Mechanical Regulation connections and nodes can be toggled off in the Network Diagram with the Show Mechanical Regulation checkbox ⑤ Regulation Numbers giving information about Internal and External connections entering a node and exiting a node (Gene) can be toggled on in the Network Diagram with the Show Regulation Number checkbox. ⑥ An Example of a different GMRN is given to showcase *DevSim's* capabilities; the example shown shows 5 genes with 6 Parallel Pathways and many connections.

(F) Edit Network Parameters (Excel)

1. Click Edit Parameters button in the GMRN panel to open Active/GeneParameters.xlsx. For each gene edit:

- Leak: basal expression
- Degradation: linear decay constant
- Initial Value: starting expression in [0,1].
- Color: name corresponding to RGB triplet used in gene plots
- Short-range mode (per gene): -2, -1, 0, +1, +2
- Long-range chemotaxis attraction and repulsion matrices (receiver x sender)

Modes +1/-1 use additive (average) adhesion logic; +2/-2 use multiplicative logic; 0 disables the short-range contribution for that gene.

Interpretation: For attraction, higher sender expression increases pull toward the sender (if +1) or reduces it (if -1). For repulsion, the sign has the analogous meaning

2. Save the workbook and return to *DevSim*; click “Refresh Diagram” to visualize the resulting genetic-mechanical channel connections.

3. Preview the GMRN, if satisfied with the setup move onto the next section.

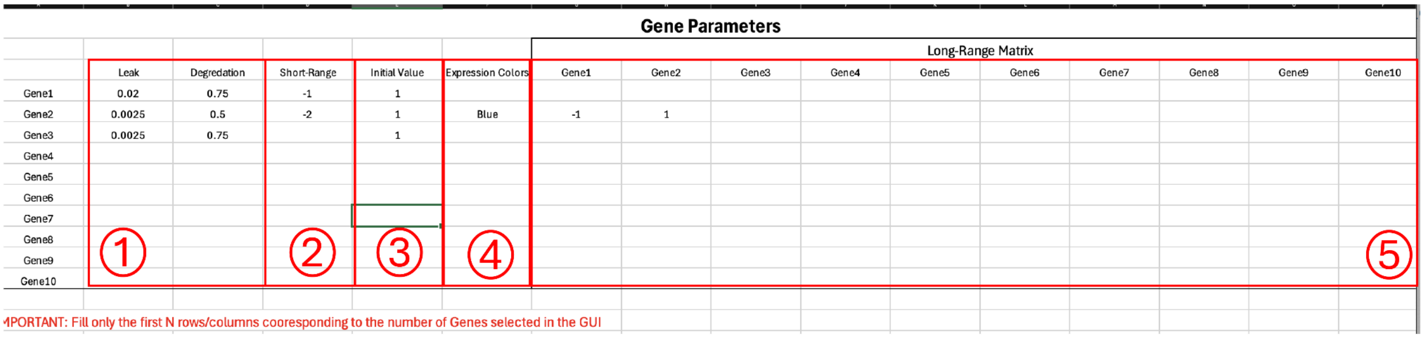
Gene Parameters

						Long-Range Matrix									
	Leak	Degredation	Short-Range	Initial Value	Expression Colors	Gene1	Gene2	Gene3	Gene4	Gene5	Gene6	Gene7	Gene8	Gene9	Gene10
Gene1	0.02	0.75	-1	1											
Gene2	0.0025	0.5	-2	1	Blue	-1	1								
Gene3	0.0025	0.75		1											
Gene4															
Gene5															
Gene6															
Gene7															
Gene8															
Gene9															
Gene10															

ɅPORTANT: Fill only the first N rows/columns cooresponding to the number of Genes selected in the GUI

Figure G7. GeneParameters.xlsx shown and can be edited to change the *DevSim* GMRN Mechanical Regulation and other simulation parameters. ① Leak (left column) and Degradation (right column) values can be changed for all genes 1 - 10. ② Short-Range force modes (-2, -1, 0, 1, 2) can be changed for each gene 1 - 10; entries in this column will be represented as gray arrows from the interacting gene(s) to A (Adhesion) to represent mechanical regulation. ③ Initial Value (from 0 - 1) can be changed for all genes 1 - 10. ④ expression color values can be changed for all genes 1 - 10 which affect the cell visualization RGB values depending on cells' gene expression values. ⑤ Long-range attraction force

modes can be changed in the Long-Range Matrix where columns are represented as the sender gene (ligand) and the rows are represented as the receiver gene; in the *DevSim* Network Diagram, entries in this matrix are represented as gray arrows from the interacting genes to C_A (Chemotaxis Attraction) to represent mechanical regulation.

(G) Run the Simulation

1. Confirm all parameters are desired for simulation. Optional: press "Save Parameters" if the user wants to store the in-GUI parameters used for the current simulation in case an error occurs, the GeneRegulatoryNetwork and GeneParameters excel files are not affected when pressing "Save Parameters" and "Load Parameters" button in the GMRN panel as the files will already be preserved if the user saved them according to the previous steps in E and F.
2. Click "Run Simulation" in the Simulation Controls panel. Doing so, *DevSim* will:

- Seed a random spherical cluster of cells.
- Delete All current OUTPUT* folders in the DevSim/ directory and create the user-defined number of new OUTPUT* folders according to how many "Total Simulations" the user selected inside of the GUI
- Create per-simulation noise in each OUTPUT folder under a "WorkSpace_Noise.mat" file
- Retrieves and unpacks the simulation parameters from the GUI and excel sheets
- Initializes the cells
- Runs the simulation via the GMRN logic, saving the cells' initial position in the respective OUTPUT folder directory under a "WorkSpace_MatrixP_Initial.csv" file; the cells' position and gene information at each time point in the simulation as "WorkSpace_Matrix_*.csv" files (the number of time points depends on dt and Tmax) then it saves the cells' final position and gene information under a "WorkSpace_Matrix_Final.csv" file.
- Once complete the Status label will read "Simulation complete!"

Live status and progress bar (simulation): When the user clicks "Run Simulation", the blue progress bar starts at 0% and the status reads "Starting simulation…" and then "Running Simulations…" when the simulations are in progress. The bar increases smoothly to 100% as batches complete. When all runs finish, the status changes to "Simulation complete!" and the bar is completely filled.

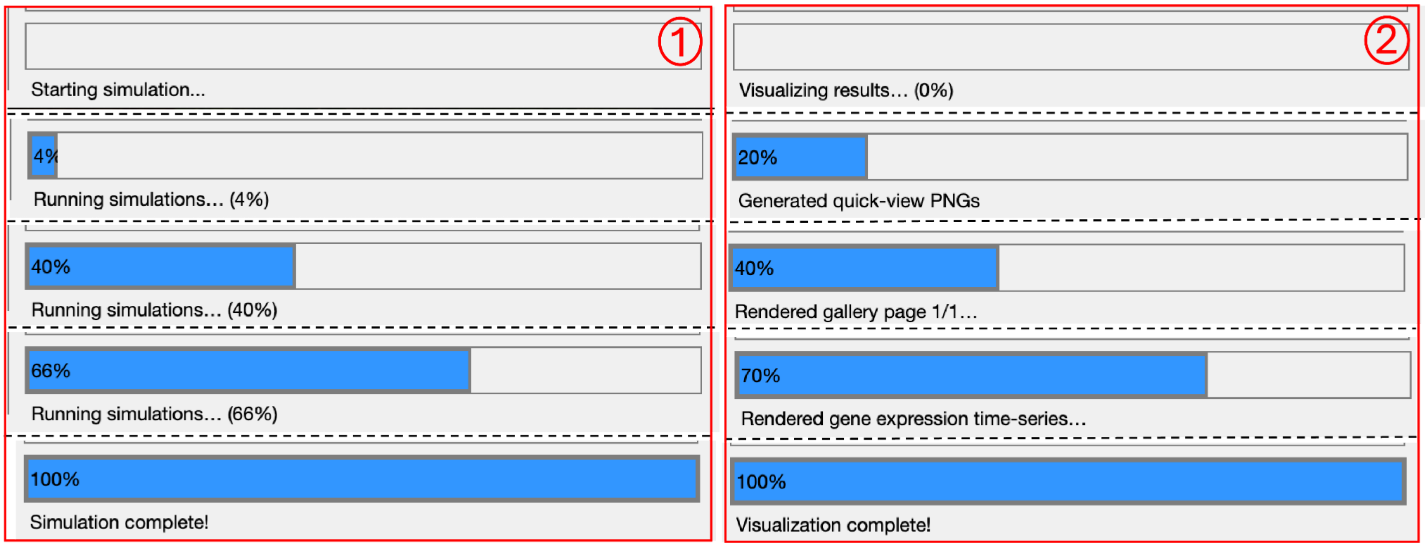

Figure G8. Run Simulation and Visualize Results Progress display. ① Column 1 shows the blue progress bar (when the user presses Run Simulation button) filling smoothly from 0% to 100% as batches complete. ② Column 2 shows the blue progress bar (when the user presses Visualize Results button) filling in stages as different visualizations complete.

(H) Visualize Results

1. Once the simulation is complete and the status label reads "Simulation complete!", click "Visualize Results" in the Visualization panel to generate the following inside of each OUTPUT directory:
   - FinalSnapshot.png per simulation (for quick viewing inside of the GUI).
   - GeneExpressionOverTime_Gene#.png (per-cell tracing in order to reveal bifurcations).
   - ShapeOverTime_*.png for 12 shape descriptors (General Sphericity, Diameter Sphericity, Intercept Sphericity, Max. Projection Sphericity, Hayakawa Roundness, Spreading Index, Elongation Ratio, Pivotability Index, Hayakawa Flatness, Wilson Flatness, Huang Shape Factor, Corey Shape Factor).
   - Gallery pages (up to 5x5 per page), will be saved in the DevSim/ directory as OUTPUT_Page#.png (multiple pages if needed).
2. Once results have been visualized and the Status label reads "Visualization complete!" inside the GUI, the first simulation should already be rendered in the GUI axes. Choose a mode in the "Visualization Mode" dropdown (Visualisation panel):
   - Quick View (PNG) - fastest; pages through 3D pre-rendered snapshots of the cells.
   - Interactive 3D View - renders a 3D scene the user can rotate, zoom, and perform other actions provided by MATLAB axis; allow for a short delay when selecting this view to load.
   - Gallery View - shows a thumbnail grid of all 3D pre-rendered cells.
3. Use the Selector (underneath the top plot located in the visualization panel):
   - In Gene Expression mode, pick Gene 1…N (shows all cells' trajectories for that gene in one simulation).
   - In Shape Descriptors mode, pick one of the 12 shape descriptions (time series for that simulation).

Auto-Visualize: enable "Auto-Visualize After Simulation" checkbox located in the visualization panel in order to immediately generate and render visualizations when a simulation finishes.

Live Status and progress bar (visualization): When "Visualize Results" is clicked, the progress bar resets to 0% and the status reads "Visualizing results…"; updates occur in steps:

- 0% → 20%: pre-render Quick View (PNG) snapshots.
- 20% → 40%: render Gallery pages
- 40% → 70%: render gene-expression time series
- 70 → 100%: render shape-descriptor time series and finalize. When finished, the status reads "Visualization complete!" and the first 3D view and graphs are automatically rendered in the Visualization panel axes.

Export Images (optional): Use the "Export Images" button (Visulization panel) to save all visulization PNGs into one parent folder in one step.

1. Press the Export Images button in the Visualization panel. A file viewer window will open, then choose a destination directory for the parent folder to be saved to, press Open to use the selected option.

2. Enter a parent folder name for this export when prompted by the popup window. *DevSim* creates <destination>/<ParentName>/

3. Inside the parent folder, *DevSim* writes OUTPUT* folders (each containing its respective PNGs) and the Gallery view PNG(s) at the top level.

4. When complete, check <ParentName>/OUTPUT*/ for per-run snapshots, gene-time series, and shape-description PNGs; check <ParentName>/ for gallery page(s).

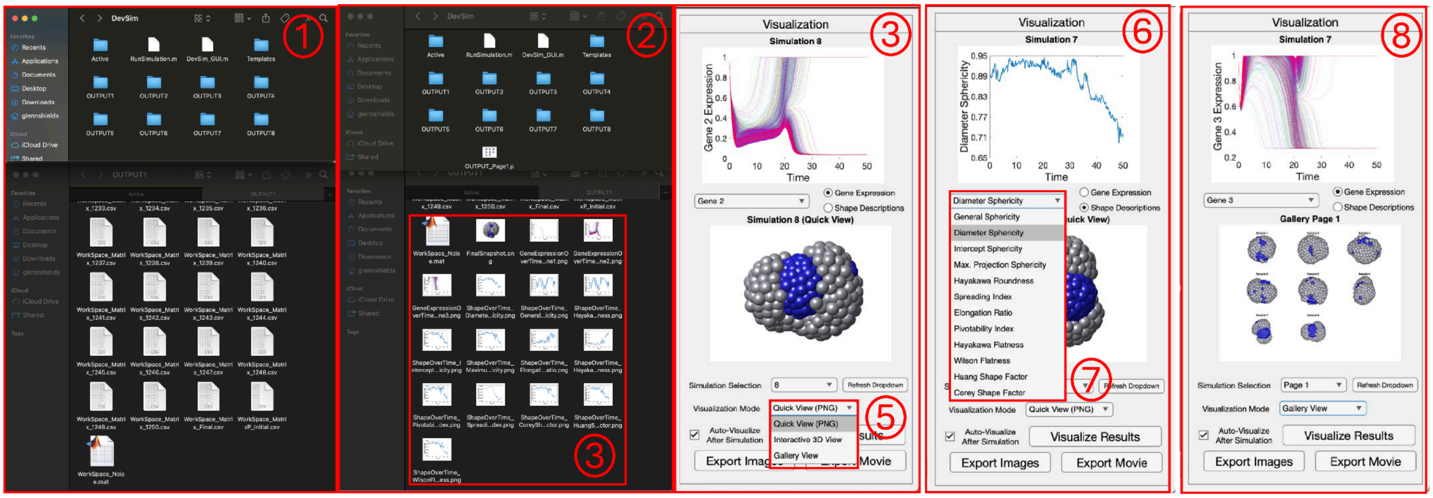

Figure G9. ① DevSim file directory after a simulation has finished is shown with the OUTPUT folders stored in the directory (top).The OUTPUT1 folder is opened as an example to show the workspace matrices and how cell positions and gene values are stored over time (bottom). ② DevSim file directory after visualization has finished is shown with the OUTPUT folders from the simulation and also the Gallery View PNG file (top). The OUTPUT1 folder is opened as an example to show what is stored after visualization ③ after visualization the 3D cell renderings, Gene expression over time, and shape descriptor over time PNG's are generated and stored within their respective OUTPUT files ④ Visualization panel shown after *DevSim* simulation has been run and the results have been visualized. Simulation 8 quick view PNG 3D image is shown in axis below and Simulation 8 Gene 2 expression over time is shown in axis above. ⑤ The 3 different visualization modes are shown in a dropdown menu. ⑥ Visualization panel shown. Simulation 7 quick view PNG 3D image is shown in axis below and Simulation 7 Diameter Sphericity Shape Descriptor over time is shown in axis above. ⑦ The 12 different shape descriptions are shown in a dropdown menu. ⑧ Visualization panel shown with Simulation 7 Gene 3 expression over time shown in axis above, and Gallery View of all 8 simulations run shown in axis below.

(I) Export Movie (optional): Use the “Export Movie” button to create an MP4 of a single simulation with user-defined time window, stride, and camera view.

1. “Click Export Movie button (Visualization panel), once pressed the Export Movie Settings popup appears, the user can then select the desired settings:

- “Output #”: choose the simulation index (e.g., 1) corresponding to an OUTPUT* folder.
- “Start time” and “End time”: any values between 0 and Tmax.
- “Frames per second (FPS)”: sets the MP4 playback FPS (does not change sampling stride).

2. "Click OK once satisfied, the Resolution (frame stride) popup appears. Choose an option to control at what resolution the movie will be created with:

- "Full (1x)": uses every saved timepoint (longest render, longest movie).
- "Half (2x), Quarter (4x), Eighth (8x)": uses every #x frames when rendering a movie (e.g. Half uses every other frame, Quarter uses every 4 frames, etc.) to shorten render/duration of movie.
- "Custom": enter any positive integer stride

3. "Click OK once satisfied, the View popup appears. Choose an option to control at what camera angle the simulation will be rendered at:

- "Top (X - Y), Front (X - Z), Side (Y - Z), Isometric"
- "Custom" - enter Azimuth (-180 to 180) and Elevation (-90 to 90)

4. Press OK and a and a file viewer window will open, choose a name for the mp4, and choose a destination directory for the MP4 file to be saved to after the rendering.

5. Press OK and the movie will start rendering. The main progress bar tracks the progress of the movie rendering, and a frame-by-frame preview window shows each rendered frame (close this at any time to cancel the movie rendering).

6. Once finished, open the directory that was chosen to and play the MP4 file to view the movie.

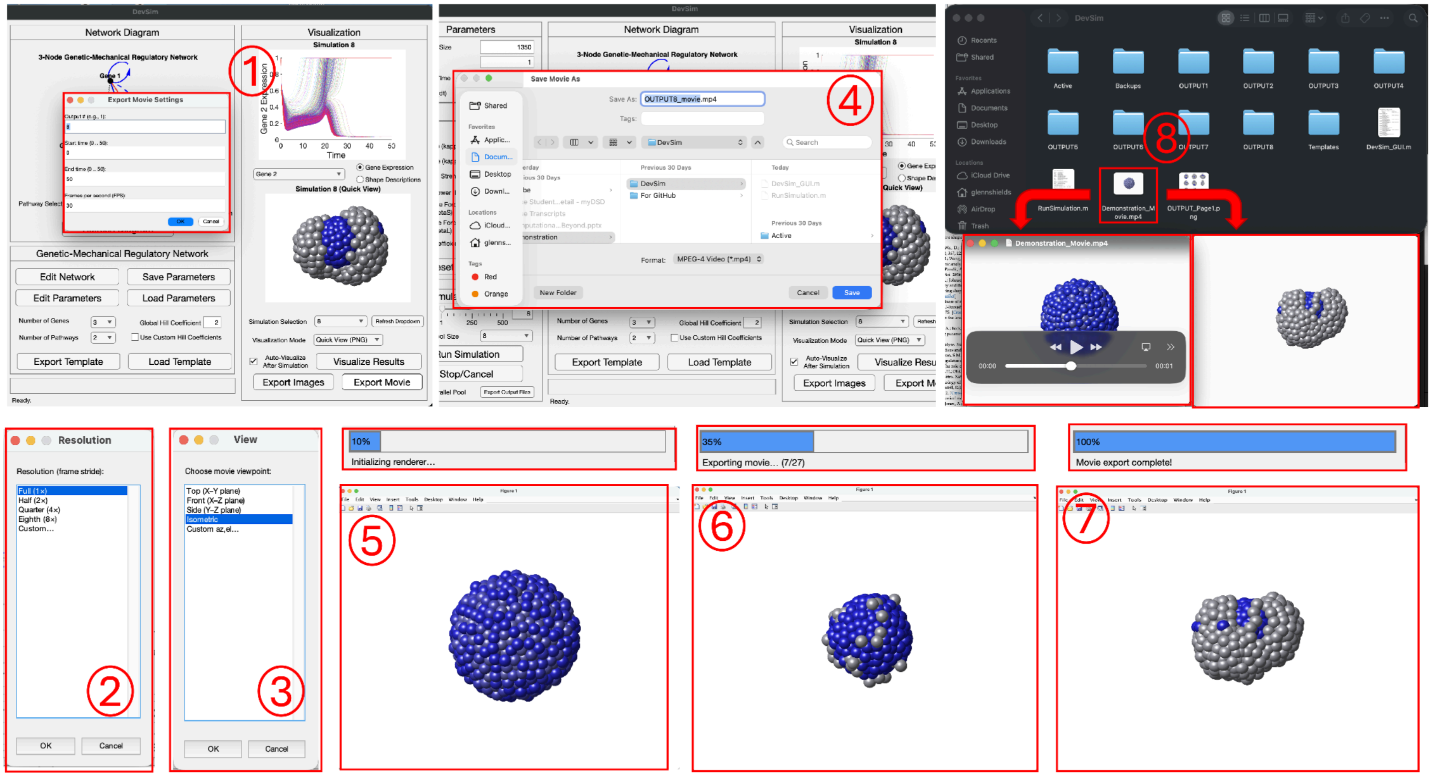

Figure G10 here to show the export movie process. ① Export Movie Settings panel shown after user presses "Export Movie" button in the Visualization panel. Users will enter the Output number (corresponding to the sample they want to generate a movie for), the start and end times, and the Frames per second. ② Once the export movie settings have been entered, an additional panel will pop up called Resolution. Here the User will either pick "Full (1x), Half (2x), Quarter(4x), Eighth(8x) or "Custom" (integer) for their movie resolution. ③ Once the resolution has been picked, another pop up window called "View" will prompt the user to pick a movie viewpoint from: "Top (X-Y plane), Front(X-Z plane), "Side (Y-Z plane), Isometric, or "Custom ax, el…". ④ Once the movie viewpoint has been chosen, a file directory viewer

will pop up, prompting the user to choose a name for their .mp4 movie file, and to choose a directory to where that .mp4 movie file will be saved to. ⑤ Once the user is finished choosing all the settings for their movie, the movie will start to render. A frame-by-frame renderer will pop on so the user can view what it actually being rendered for their movie in real time which is shown via the bottom red box. The top red box shows the progress bar updates in *DevSim* as the movie is rendering. ⑥ Another timepoint choosing different rendering states in the movie that will be shown to the user (bottom red box). The top red box shows the progress bar as well as the status label showing useful information regarding the movie rendering. The (7/27) number shows that the movie has rendered 7 out of its 27 frames. ⑦ The final rendering timepoint and progress bar are shown after the movie has finished rendering. ⑧The file directory that the .mp4 file was saved to is shown and the movie file is highlighted in red. Underneath are two instances of the same movie file open, the left one shows that it is indeed a playable .mp4 movie during an initial frame of the simulation; the right one shows a final frame of the movie.

(J) Import precomputed OUTPUT folders (Refresh Dropdown Option)

DevSim's simulation and visualization are modular. If the user already have *DevSim*-compatible OUTPUT folders (e.g., OUTPUT1/ , OUTPUT2/ …); the user can load and visualize them without re-running simulations.

1. Prepare the *DevSim* folder: In DevSim/ directory, remove or move out any existing OUTPUT* folders to avoid conflicts.
2. Copy in results: Place the precomputed OUTPUT* folders directly under DevSim/ directory.
3. Open DevSim if not already running, and go to Visualization panel
4. Click the "Refresh" dropdown (Visualization panel). *DevSim* scans the DevSim/ directory, detects the imported OUTPUT* folders, and updates the "Simulation Selection" dropdown to match the count.
5. If PNG images are already present, choose a simulation in "Simulation Selection"; views render immediately.
6. If the imported results have not been visualized yet (no PNGs), set the "Total Simulations" box in Simulation Controls to the total number of OUTPUT folders that were imported into the DevSim/ directory. Then click "Visualize Results" to generate them and then follow instructions in section H from step 2 accordingly.

(K) Save/load parameters and templates

1. Save Parameters: writes current GUI parameter values to Active/UserParams.xlsx.
2. Load Parameters: reads Active/UserParams.xlsx into the GUI (useful when reopening *DevSim*)
3. Export Template: packages the trio (UserParams.xlsx, GeneRegulatoryNetwork.xlsx, GeneParameters.xlsx) into Templates/<name>/.
4. Load Template: copies a chosen template pack into Active/ (warning: if loading a template the current Active files will be replaced; the GUI will prompt and warn the user before this happens)

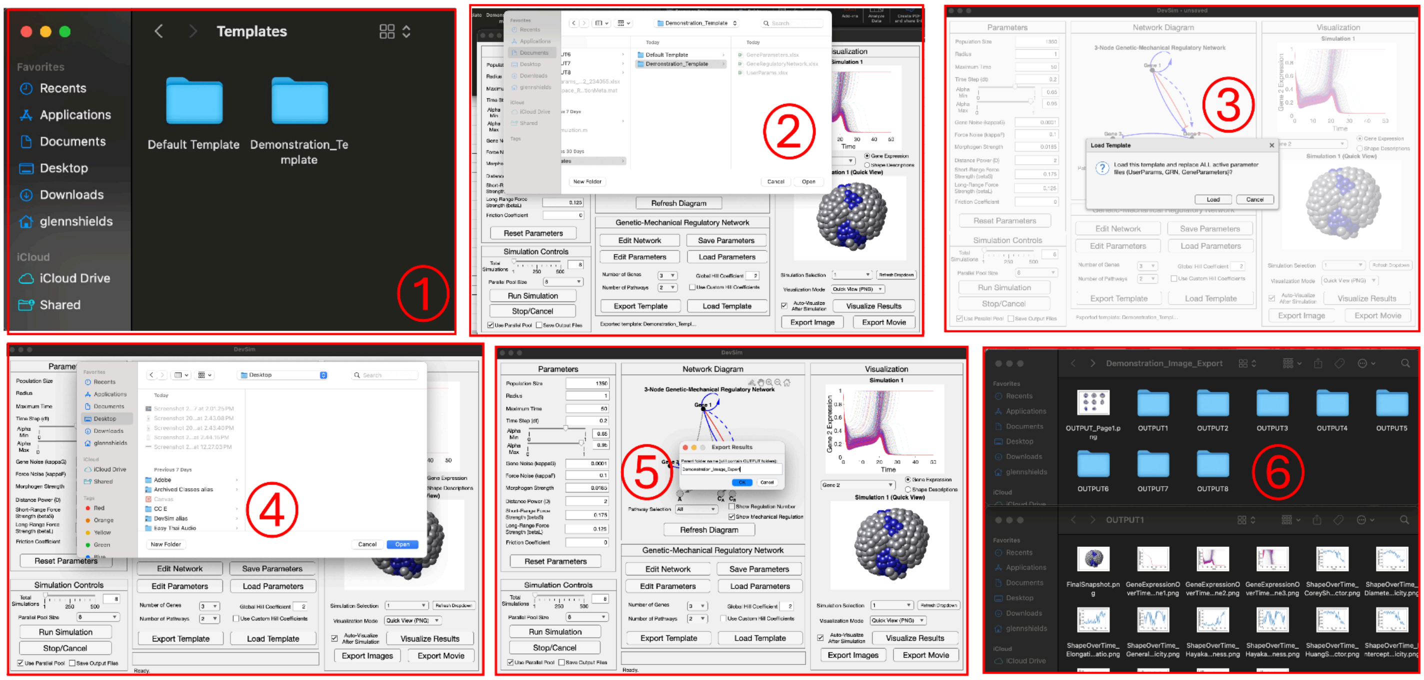

Figure G11. ① Templates folder inside of DevSim directory is shown with the Default Template and a Demonstration Template created via pressing the Load Template button in the GMRN panel inside of *DevSim* GUI ② Shows the file directory users are shown after pressing the "Load Template" button; once a template is selected and the user presses "Open" in the file directory ③ *DevSim* will prompt the user to confirm that they want to load the selected template and as a result, replace all active parameter files where users can either go forward with the process by pressing Load or stop it by pressing Cancel. ④ Shows the file directory window users will be sent to once pressing Export Images Button in the Visualization panel inside of *DevSim* GUI. The users will select the location they want to save the parent folder holding all the .png files from *DevSim*'s visualize results feature. ⑤ Once Users select a location for the parent folder they will be prompted to name the parent folder which will hold all the .png files in their respective OUTPUT folders ⑥ Parent folder created via Export Images button is shown for demonstration purposes called "Demonstration_Image_Export", within the directory there is the gallery view .png file, and the OUTPUT folders (above). An OUTPUT1 folder within the parent folder is opened and all of the .png files that *DevSim* created via the Visualize Results function are stored inside (below).

(L) Export Output Files: use this feature to bundle an entire experiment into a single parent folder for archiving or sharing. Inside of the parent folder will include all the information for the experiment including the OUTPUT folders and all their contents, the gallery PNG's, and the Active folder containing the simulation settings that were used for that Simulation.

1. Press the Export Output Files button in the Simulation Controls panel. A file viewer window will open, then choose a destination directory for the parent folder to be saved to, press Open to use the selected option.

2. Enter a parent folder name for this export when prompted by the popup window. *DevSim* creates <destination>/<ParentName>/

3. Inside the parent folder, *DevSim* copies all OUTPUT* folders and their contents along with the Active folder containing the current simulation parameters, and the Gallery view PNG(s) saved at the top level.

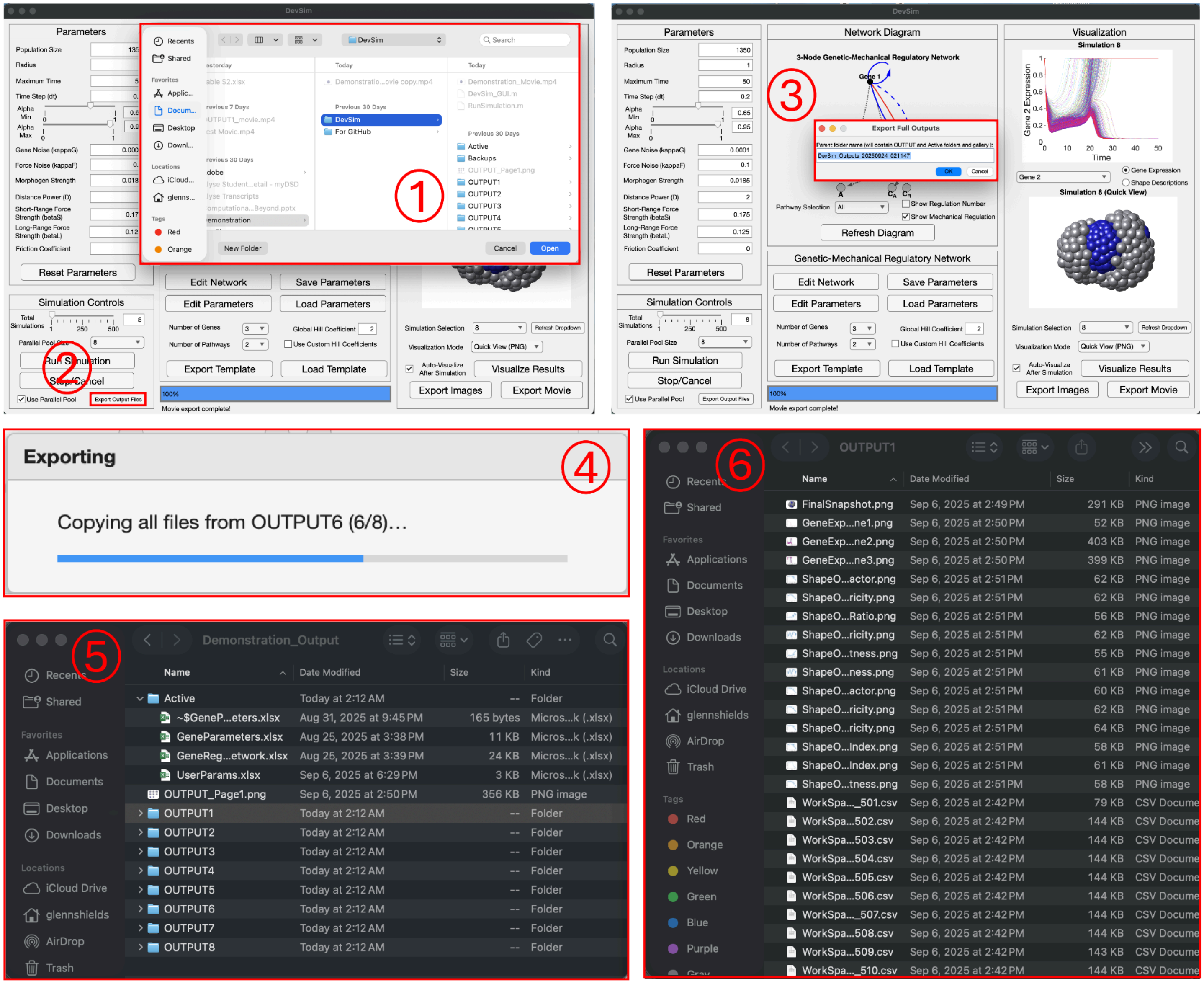

Figure G12 Here showing export output files process. ① Shows the file directory Users are shown after pressing "Export Output Files Button". ② Export Output Files button located in the bottom of the Simulation Controls Panel that users will press to start the Export Output Files function. ③ Once users select a location for the parent folder, they will be prompted to name the parent folder which will hold all of the OUTPUT* folders, the Active folder, and the gallery view .png files. ④ Once the user selects the name for the parent folder, an exporting panel pop up showing the progress of copying all the OUTPUT folders into the Parent folder is shown. ⑤ Parent folder that was created for the demonstration and some of its contents are displayed for reference. ⑥ OUTPUT1 folder inside of the Parent folder that was created for the demonstration is displayed for reference.

Parameter Glossary (quick)

Population: number of coarse-grained cells

Radius: Sets initial spacing scale and interaction distances

Maximum Time: total simulated time (in arbitrary units)

Time Step (dt): integration step size (smaller dt → more time points).

Alpha Max: Sets the upper bound for the typical balancing spacing in between cells

Alpha Min: Sets the lower bound for the typical balancing spacing in between cells

Gene Noise: Sets the gene noise amplitude

Force Noise: Sets the force noise amplitude

Morphogen Strength: Scales the external signal influence

Distance Power: Sets distance decay for external/morphogen signaling interactions

Short-Range Force Strength: Sets the short-range force amplitude

Long-Range Force Strength: Sets the long-range force amplitude

Friction Coefficient: Overdamped environment drag coefficient

Troubleshooting (quick)

- *DevSim* Folder is downloaded and the GUI is open but none of the Excel files can be accessed via “Edit Network” or “Edit Parameters” buttons, and features inside of the GUI don't work
  Make sure in the MATLAB window that the current folder is DevSim root, if not none of the features in *DevSim* will work properly. After setting the folder root, close the *DevSim* app and reopen it in MATLAB under the correct directory, then retry features.
- Diagram looks empty or wrong
  Click Refresh Diagram. Confirm “Number of Genes” and “Number of Pathways” in the GUI match the edited Excel ranges. Make sure Excel was saved before refreshing diagram inside the GUI
- Visualization panel is blank
  Press the “Refresh Dropdown” button, then select any simulation under the “Simulation Selection” dropdown. If there are no rendered images, most likely the visualization did not generate or save correctly. Press Visualize Results and wait for visualization to complete.
