# Supplementary material for: Cooperative short- and long-range interactions enable robust symmetry breaking and axis formation
Source: bioRxiv. 2025 Sep 29:2025.09.27.678924. Preprint. [Version 1] doi: 10.1101/2025.09.27.678924 (PMC12621816; doi:10.1101/2025.09.27.678924)
Supplement: Supplement 12 [file media-12.pdf]

METHOD DETAILS — Biophysical model (integrated description)

High-level overview. Each simulated cell carries a small gene network that (i) integrates *intracellular* regulations among its own genes and (ii) aggregates *intercellular* signals that decay with distance. The resulting gene state is then *read out* into two mechanical knobs: a symmetric short-range parameter $\alpha_{m,n}$ (used by the contact force) and a directed long-range parameter $\beta_{m,n}$ (used by the distance force). Cells move in an overdamped medium under these forces plus noise. A minimal timer → bifurcation → lock-in program ensures that mechanics turn on only after a transient gene stage, allowing a homogeneous population to self-organize an axis. Below, we present the exact mathematics used in the model and interleave concise explanations so the logic is clear. (All equations and symbols match the METHOD DETAILS — Biophysical model (condensed formulas) and the force definitions shown in the main text figures.)

State

There are N_g genes programmed within N_c cells. For cell m, the N_g-vector of gene expressions is

$$\mathbf{G}_m \;=\; (G_{m,1}, \ldots, G_{m,N_g})^\top \in [0,1]^{N_g},$$

initialized at either 0 or 1. Gene and position noises $\boldsymbol{\xi}_G$ and $\boldsymbol{\xi}_r$ are zero-mean. Cells have positions $\mathbf{r}_m \in \mathbb{R}^3$; pair geometry uses

$$\mathbf{d}_{m,n} = \mathbf{r}_m - \mathbf{r}_n, \quad d_{m,n} = \|\mathbf{d}_{m,n}\|, \quad \hat{\mathbf{d}}_{m,n} = \mathbf{d}_{m,n}/d_{m,n}.$$

We use a three-tensor $\mathbf{B}^{(\text{intra})} \in \{-1, 0, +1\}^{N_g \times N_g \times N_p}$ to encode intracellular regulation (activation +1, inhibition −1, no effect 0); each slice p is a parallel pathway, rows are targets, columns regulators. Analogously, $\mathbf{B}^{(\text{inter})}$ encodes intercellular regulation. "Sigmoid/Hill" ("Sigmod") nonlinearities are applied component-wise as specified below.

Intracellular regulation (Eq. 1; what it does).

The intracellular block converts current gene levels in cell m into an effective regulatory output $\mathbf{R}_m^{(\text{intra})}$. Responsive concentration and sensitivity vectors are $\mathbf{K}^{(\text{intra})}$ and $\mathbf{H}^{(\text{intra})}$. The exact form is

$$\boxed{\mathbf{R}_m^{(\text{intra})} \;=\; \mathbf{1} \;+\; \operatorname{sign}\!\big\{\mathbf{B}^{(\text{intra})}\big\} \;\circ\; \left[\sigma_{\mathbf{H}^{(\text{intra})}}\!\big(\mathbf{G}_m - \mathbf{K}^{(\text{intra})}\big) \;-\; \frac{\mathbf{1}}{\|\mathbf{B}^{(\text{intra})}\|_1}\right]} \tag{1}$$

where $\circ$ is the Hadamard product, $\mathbf{1}$ is an all-ones vector of the needed size, $\|\cdot\|_1$ is the element-sum of absolute entries, and $\sigma_{\mathbf{H}}$ applies a component-wise sigmoid/Hill with sensitivity $\mathbf{H}$ and threshold $\mathbf{K}$. *Interpretation:* the sign mask gates activation/inhibition; the sigmoid maps inputs into $[0,1]$; the small constant recenters the response so "no net input" is neutral.

Intercellular regulation (Eq. 2; what it does).

Intercellular regulation aggregates morphogen-like inputs from all other cells with a power-law distance decay. With responsive concentration $\mathbf{K}^{(\text{inter})}$, sensitivity $\mathbf{H}^{(\text{inter})}$, morphogen production M, diffusion exponent n, and cell radius l,

$$\boxed{\mathbf{R}_m^{(\text{inter})} \;=\; \mathbf{1} \;+\; \operatorname{sign}\!\big\{\mathbf{B}^{(\text{inter})}\big\} \;\circ\; \left[\sigma_{\mathbf{H}^{(\text{inter})}}\!\big(\mathbf{S}_m - \mathbf{K}^{(\text{inter})}\big) \;-\; \frac{\mathbf{1}}{\|\mathbf{B}^{(\text{inter})}\|_1}\right], \qquad \mathbf{S}_m \;=\; \sum_{n \neq m} M \, \frac{\mathbf{G}_n}{\left(\frac{d_{m,n}}{l}\right)^D}} \tag{2}$$

Interpretation: $\mathbf{S}_m$ is a power-law average of neighbors' gene outputs $(l/d)^n$, giving nearer cells more weight. Passing $\mathbf{S}_m$ through the same sign/sigmoid template yields an intercellular regulatory output.

Gene → mechanics readouts and forces (Eqs. 3–4; what they do).

Gene state sets two mechanical channels that the force laws consume.

Short-range channel and force (Eq. 3). The short-range readout is symmetric in the pair and is gated by the timer $G1$. With constant K_α,

$$\boxed{\alpha_{m,n} \;=\; 0.95 \;-\; K_\alpha\,(1-G1_m)(1-G1_n)\,\frac{1-G2_m+G2_n}{2}} \tag{3}$$

The short-range force (used only when $d_{m,n} < 2l$) is exactly

$$\boxed{\mathbf{F}^{\text{short}}_{m,n} \;=\; \left(1-\frac{d_{m,n}}{2\,l\,\alpha_{m,n}}\right)\hat{\mathbf{d}}_{m,n}, \qquad d_{m,n} < 2l} \tag{4}$$

Interpretation: near contact the force can be adhesive (positive along $\hat{\mathbf{d}}_{m,n}$); very small separations become effectively repulsive; the balance is set by $\alpha_{m,n}$, which only changes once $G1$ drops.

Long-range channel and force (Eq. 4). The directed long-range readout depends only on the *signaling* cell n; with constant K_β,

$$\boxed{\beta_{m,n} \;=\; K_\beta\,(1-G1_n)\,G2_n} \tag{5}$$

The long-range force (used only when $d_{m,n} > 2l$) is

$$\boxed{\mathbf{F}^{\text{long}}_{m,n} \;=\; \left(-\frac{\beta_{m,n}}{d^2_{m,n}}\right)\hat{\mathbf{d}}_{m,n}, \qquad d_{m,n} > 2l} \tag{6}$$

Interpretation: a sender with low $G1$ and high $G2$ exerts a $1/d^2$ pull on neighbors; the sign convention for attraction/repulsion is as in the figure.

Discrete-time updates (Eq. 5; how a step proceeds).

Let $\mathbf{D}$ be degradation, $\mathbf{L}$ promoter leakage. At each time step Δt,

$$\boxed{\mathbf{G}(T+\Delta T) \;=\; \mathbf{G}(T) \;+\; \boldsymbol{\xi}_G \;+\; \Delta T\Big[\exp\big\{\big(\mathbf{R}^{(\text{intra})}\!\cdot\!\mathbf{R}^{(\text{inter})}\big)\big\}\,\mathbf{1} \;-\; \mathbf{D}\odot\mathbf{G}(T) \;+\; \mathbf{L}\Big]} \tag{7}$$

$$\boxed{\mathbf{r}(T+\Delta T) \;=\; \mathbf{r}(T) \;+\; \frac{1}{\eta}\boldsymbol{\xi}_r \;+\; \frac{\Delta T}{\eta}\Big[\sum_{n\neq m}\mathbf{F}^{\text{short}}_{m,n} \;+\; \sum_{n\neq m}\mathbf{F}^{\text{long}}_{m,n}\Big]} \tag{8}$$

Interpretation: (i) build intercellular inputs $\mathbf{S}_m$; (ii) compute $\mathbf{R}^{(\text{intra})}_m$ and $\mathbf{R}^{(\text{inter})}_m$; (iii) update genes by production (the exponential of the product of regulatory matrices, times $\mathbf{1}$), minus degradation plus leakage and noise; (iv) form α, β via the exact readouts; (v) sum pair forces and advance positions with noise.

Practical update order (one glance).

1. Compute $\mathbf{S}_m = \sum_{n \neq m} M\, \mathbf{G}_n / (\frac{d_{m,n}}{l})^D$.
2. Compute $\mathbf{R}_m^{(\text{intra})}$ by Eq. (1); $\mathbf{R}_m^{(\text{inter})}$ by Eq. (2).
3. Update $\mathbf{G}$ by Eq. (7) (clip to $[0, 1]$).
4. For each pair, compute $\alpha_{m,n}$ by Eq. (3) and $\beta_{m,n}$ by Eq. (5).
5. For each pair, apply Eq. (4) if $d_{m,n} < 2l$ and Eq. (6) if $d_{m,n} > 2l$.
6. Update positions by Eq. (8).

Default parameter values (as specified in the Methods document)

Table 1 reproduces the default parameters and values.

Table 1: Default of system parameter and value assignment.

Biological significance	System parameter	Value assignment / remark
Cell radius	l	1
Short-range force (relative balanced distance between neighboring cells)	α	—
Long-range force	β	—
Responsive strength of short-range force	K_α	0.175
Responsive strength of long-range force	K_β	0.125
Strength of morphogen	M	0.0185
Distribution of morphogen (diffusion and degradation)	D	2
Responsive concentration of intracellular regulation	$R^{(intra)}$	—
Responsive concentration of intercellular regulation	$R^{(inter)}$	—
Responsive sensitivity of intracellular regulation	$H^{(intra)}$	2
Responsive sensitivity of intercellular regulation	$H^{(inter)}$	2
Time step	ΔT	0.2
Time duration	T_{total}	150
Environmental viscosity	η	1
Random noise on gene expression	κ_G	0.0001
Random noise on cell position	κ_r	0.1

What the three-gene motif is doing (intuitive map). $G1$ is a *timer/gate*: while $G1$ is high, $(1-G1)$ terms in Eqs. (3)–(5) keep α, β from responding. As $G1$ decays, mechanics switch on. $G2$ and $G3$ form a mutual-inhibition toggle (bifurcation) that partitions cells during the gene-establishment stage; in maintenance, this partition is stabilized and then α and β drive the

mechanical reorganization. The long-range pulls *coherently order the periphery* while the short-range channel maintains cohesion where needed, producing a single axis from homogeneous initial conditions.

METHOD DETAILS — Biophysical model (condensed formulas)

When coupled with gene regulation, a coarse-grained model can incorporate and reproduce a lot of details observed in the real developmental biological systems [Delile et al. *Nat. Commun.* 2017; Nissen et al. *PLoS Biol.* 2017]. The model setting in this project is introduced below.

- There are N_g genes programmed within N_c cells, with their expression levels represented by the vector $\boldsymbol{G} = \left\{G_1; \dots; G_{N_g}\right\}$, ranging from 0 to 1, either of which can be the initial value. The genetic regulatory network is represented by an $N_g \times N_g \times N_p$ matrix $\boldsymbol{B}_I = \left\{B_{I(g_1,g_2,p)}\right\}$, where $B_{I(g_1,g_2,p)} = -1,1,0$ indicates inhibition, activation, or no effect of gene g_2 on gene g_1, and the combination of regulatory terms based on Sigmod functions determines the whole regulatory network [Dibaeinia et al. *Nucleic Acids Res.* 2021]. The 2D matrices for each p $\left(\text{where } p = 1,2,3,\dots,N_p\right)$ represent parallel pathways, while each 2D matrix within a p describes a series pathway. For a specific cell:
    - Using the responsive concentration $\boldsymbol{K}_I$ and sensitivity $\boldsymbol{H}_I$, the intracellular regulatory matrix is
$$\boldsymbol{R}_I = 1 + sign\{\boldsymbol{B}_I\}\left(\frac{1}{2} - \frac{\boldsymbol{K}_I^{\boldsymbol{H}_I}}{\boldsymbol{K}_I^{\boldsymbol{H}_I} + \boldsymbol{G}^{\boldsymbol{H}_I} \cdot \boldsymbol{1}_{1\times N_g}}\right) - \frac{sign\{|\boldsymbol{B}_I|\}}{2} \qquad (1)$$
    - Similarly, the intercellular signaling regulatory network is represented by an $N_g \times N_g \times N_p$ matrix $\boldsymbol{B}_E = \left\{B_{E(g_1,g_2,p)}\right\}$. With responsive concentration $\boldsymbol{K}_E$ and sensitivity $\boldsymbol{H}_E$, morphogen production $\boldsymbol{M}$, diffusion level $\boldsymbol{D}$, and cell radius $\boldsymbol{l}$, the extracellular regulatory matrix is
$$\boldsymbol{R}_E = \frac{1}{2} + sign\{\boldsymbol{B}_E\}\left(\frac{1}{2} - \frac{\boldsymbol{K}_E^{\boldsymbol{H}_E}}{\boldsymbol{K}_E^{\boldsymbol{H}_E} + \boldsymbol{S}^{\boldsymbol{H}_E} \cdot \boldsymbol{1}_{1\times N_g}}\right) - \frac{sign\{|\boldsymbol{B}_E|\}}{2}, \boldsymbol{S} = \sum^{N_c}{}_{\substack{c_2 \neq c_1 \\ |\boldsymbol{r}_{c_2} - \boldsymbol{r}_{c_1}| > l_{c_2} + l_{c_1}}} \frac{\boldsymbol{M}\cdot\boldsymbol{G}_{c_2}}{|\boldsymbol{r}_{c_2} - \boldsymbol{r}_{c_1}|^{\boldsymbol{D}}} \qquad (2)$$
    - The adhesion coefficient α_{c_1,c_2} and short-range force $\boldsymbol{F}_{S(c_1)}$ are regulated by a $1 \times N_g$ vector $\boldsymbol{B}_S$:
$$\begin{cases} \boldsymbol{R}_S = 1 + sign\{\boldsymbol{B}_S\}\left(\frac{1}{2} - \boldsymbol{G}\right) - \frac{sign\{|\boldsymbol{B}_S|\}}{2}, \alpha_{c_1,c_2} = 0.95 - K_\alpha e^{ln\{\boldsymbol{R}_{S(c_1)} \cdot \boldsymbol{R}_{S(c_2)}\} \cdot \boldsymbol{1}_{N_g\times 1}} \\ \boldsymbol{F}_{s,c_1} = \sum^{N_c}{}_{\substack{c_2 \neq c_1 \\ |\boldsymbol{r}_{c_2} - \boldsymbol{r}_{c_1}| < l_{c_2} + l_{c_1}}} \left[1 - \frac{|\boldsymbol{r}_{c_2} - \boldsymbol{r}_{c_1}|}{\alpha_{c_1,c_2}(l_{c_2} + l_{c_1})}\right] \cdot \frac{\boldsymbol{r}_{c_2} - \boldsymbol{r}_{c_1}}{|\boldsymbol{r}_{c_2} - \boldsymbol{r}_{c_1}|} \end{cases} \qquad (3)$$
    - The chemotactic coefficient $\boldsymbol{\beta}_{c_1,c_2}$ and long-range force $\boldsymbol{F}_{L(c_1)}$ are regulated by an $N_g \times N_g$ matrix $\boldsymbol{B}_L$:
$$\begin{cases} \boldsymbol{R}_L = 1 + sign\{\boldsymbol{B}_L\}\left(\frac{1}{2} - \boldsymbol{G}\boldsymbol{1}_{1\times N_g}\right) - \frac{sign\{|\boldsymbol{B}_L|\}}{2}, \boldsymbol{\beta}_{c_1,c_2} = K_\beta \left[\frac{\boldsymbol{M}\cdot\boldsymbol{G}_{c_2}}{|\boldsymbol{r}_{c_2} - \boldsymbol{r}_{c_1}|^{\boldsymbol{D}+1}}\right] \cdot \frac{\boldsymbol{r}_{c_2} - \boldsymbol{r}_{c_1}}{|\boldsymbol{r}_{c_2} - \boldsymbol{r}_{c_1}|} \\ \boldsymbol{F}_{L,c_1} = \sum^{N_c}{}_{\substack{c_2 \neq c_1 \\ |\boldsymbol{r}_{c_2} - \boldsymbol{r}_{c_1}| > l_{c_2} + l_{c_1}}} \left[\boldsymbol{\beta}_{c_1,c_2} \cdot \boldsymbol{1}_{1\times N_g} \cdot \boldsymbol{R}_L \cdot \boldsymbol{1}_{N_g\times 1}\right]^T \cdot \boldsymbol{1}_{N_g\times 1} \end{cases} \qquad (4)$$
    - Finally, with degradation coefficient $\boldsymbol{D}$, promotor leakage $\boldsymbol{L}$, gene expression noise $\boldsymbol{\xi}_G = \sqrt{\Delta T}\kappa_G \cdot Gaussian\ (Mean = 1, Standard\ Deviation = 1)$, and cell position noise $\boldsymbol{\xi}_r = \sqrt{\Delta T}\kappa_r \cdot Gaussian\ (Mean = 1, Standard\ Deviation = 1)$, the gene expression $\boldsymbol{G}$ and cell position $\boldsymbol{r}$ evolve according to:
$$\begin{cases} \boldsymbol{G}(T + \Delta T) = \boldsymbol{G}(T) + \boldsymbol{\xi}_G + \Delta T\left(\sum_{p=1}^{N_p} e^{ln\{\boldsymbol{R}_I \cdot \boldsymbol{R}_E\} \cdot \boldsymbol{1}_{N_g\times 1}} - \boldsymbol{D} \cdot \boldsymbol{G} + \boldsymbol{L}\right) \\ \boldsymbol{r}(T + \Delta T) = \boldsymbol{r}(T) + \frac{1}{\eta}\boldsymbol{\xi}_r + \frac{\Delta T}{\eta}\left[\sum_{p=1}^{N_p} \boldsymbol{F}_S + \sum_{p=1}^{N_p} \boldsymbol{F}_L\right] \end{cases} \qquad (5)$$

All the modeling for initial cell position generation (Figure S2), simulation of pre-differentiated two cell types interacting mechanically with symmetry breaking (Figure 4), and simulation of genetic-mechanical regulatory network with symmetry breaking, are implemented on *Matlab* [Natick, Massachusetts: The MathWorks Inc. 2024].

In principle, the interaction between genes and between cells, as well as between genes and cells, can be encoded extensively, allowing researchers to explore all kinds of problem related to molecular-to-multicellular self-organization, including but not limited to fundamental questions (design principle regarding physical and mathematical structures [Ma et al. *Cell* 2009; Chau et al. *Cell* 2012]), synthetic systems (*synNotch*, *synCAM*, and others [Toda et al. *Science* 2018; Stevens et al. *Nature* 2022]), and manipulation of the real ones (human embryoid in normal experimental conditions as well as the ones mimicking desease which need to be "cured" [Moris et al. *Nature* 2020; Arias et al. *Dev. Biol.* 2022]).
